# The ATPase SRCAP is associated with the mitotic apparatus, uncovering novel molecular aspects of Floating-Harbor syndrome

**DOI:** 10.1186/s12915-021-01109-x

**Published:** 2021-09-02

**Authors:** Giovanni Messina, Yuri Prozzillo, Francesca Delle Monache, Maria Virginia Santopietro, Maria Teresa Atterrato, Patrizio Dimitri

**Affiliations:** 1 Dipartimento di Biologia e Biotecnologie “Charles Darwin” Sapienza Università di Roma, Via dei Sardi, 70, Roma, Italy; 2 Istituto Pasteur Italia Fondazione Cenci-Bolognetti, Viale Regina Elena, 291, 00161 Roma, Italy

**Keywords:** Floating-Harbor, SRCAP, Cell cycle, Cytokinesis regulators, Midbody

## Abstract

**Background:**

A variety of human genetic diseases is known to be caused by mutations in genes encoding chromatin factors and epigenetic regulators, such as DNA or histone modifying enzymes and members of ATP-dependent chromatin remodeling complexes. Floating-Harbor syndrome is a rare genetic disease affecting human development caused by dominant truncating mutations in the SRCAP gene, which encodes the ATPase SRCAP, the core catalytic subunit of the homonymous chromatin-remodeling complex. The main function of the SRCAP complex is to promote the exchange of histone H2A with the H2A.Z variant. According to the canonical role played by the SRCAP protein in epigenetic regulation, the Floating-Harbor syndrome is thought to be a consequence of chromatin perturbations. However, additional potential physiological functions of SRCAP have not been sufficiently explored.

**Results:**

We combined cell biology, reverse genetics, and biochemical approaches to study the subcellular localization of the SRCAP protein and assess its involvement in cell cycle progression in HeLa cells. Surprisingly, we found that SRCAP associates with components of the mitotic apparatus (centrosomes, spindle, midbody), interacts with a plethora of cytokinesis regulators, and positively regulates their recruitment to the midbody. Remarkably, SRCAP depletion perturbs both mitosis and cytokinesis. Similarly, DOM-A, the functional SRCAP orthologue in *Drosophila melanogaster*, is found at centrosomes and the midbody in Drosophila cells, and its depletion similarly affects both mitosis and cytokinesis.

**Conclusions:**

Our findings provide first evidence suggesting that SRCAP plays previously undetected and evolutionarily conserved roles in cell division, independent of its functions in chromatin regulation. SRCAP may participate in two different steps of cell division: by ensuring proper chromosome segregation during mitosis and midbody function during cytokinesis. Moreover, our findings emphasize a surprising scenario whereby alterations in cell division produced by SRCAP mutations may contribute to the onset of Floating-Harbor syndrome.

**Supplementary Information:**

The online version contains supplementary material available at 10.1186/s12915-021-01109-x.

## Background

In the last two decades, it has been shown that mutations in genes encoding a variety of chromatin factors and epigenetic regulators, such as DNA or histone modifying enzymes and members of ATP-dependent chromatin remodeling complexes, are crucial players in human genetic diseases and cancer [1–4]. Floating-Harbor syndrome (FHS), also known as Pelletier–Leisti syndrome [MIM number 136140], is a human developmental disorder characterized by delayed bone mineralization and growth deficiency, which are often associated with intellectual disability and skeletal and craniofacial abnormalities [5–8].

*SRCAP* (SNF2-related CBP activator protein) is the causative gene of FHS [6–8]. It maps to chromosome 16p11.2 and is predicted to undergo alternative splicing giving rise to three putative isoforms of about 343, 337, and 327 kD (https://www.uniprot.org/uniprot/Q6ZRS2). The full-length isoform corresponds to the ATPase catalytic subunit of the homonymous multiprotein chromatin-remodeling complex [9], while the shorter variants have not yet been investigated.

The SRCAP complex is member of the evolutionarily conserved INO80 family of ATP-dependent chromatin remodeling complexes and contains a dozen subunits [9–17]. The primary function of SRCAP complex is to catalyze the exchange of canonical histone H2A with the H2A.Z variant [9, 18, 19].

FHS has a dominant inheritance pattern caused by nonsense or frameshift mutations in exons 33 and 34 of *Srcap* gene [6]. These mutations are supposed to produce a C-terminal-truncated SRCAP protein variant missing the AT-hook motifs with DNA-binding activity and are possibly responsible for a dominant negative effect triggering the onset of FHS [6, 8]. Recently, localization assays in human embryonic cranial neural crest cells showed that overexpressed GFP/Flag-tagged versions of C-terminal-truncated SRCAP are largely excluded from the nucleus but present in the cytoplasm [20], suggesting that FHS mutations affect the nuclear localization of SRCAP. The SRCAP protein can also function as a transcriptional activator by binding to the cAMP response element-binding protein (CREB)-binding protein (CREBBP or CBP) [21]. Finally, a role of SRCAP in DNA-end resection was also proposed [22].

The *Drosophila melanogaster domino* gene is orthologous to human *SRCAP* [23, 24]. It encodes two isoforms, DOM-A and DOM-B, the latter carrying a shorter C-terminal region. DOM-A was originally found to be the main subunit of the *Drosophila* Tip60 (dTip60) chromatin-remodeling complex [25] whose subunits share high sequence identity and functional conservation with SRCAP and p400/Tip60 human complexes [10]. Recently, DOM-A and DOM-B were suggested to define two different chromatin remodeling complexes, called DOM-A.C and DOM-B.C, characterized by different functions and subunit compositions [26]. Interestingly*, domino* lethal alleles are recessive and result in developmental arrest at third instar larval stage before pupation [23], while SRCAP lethal alleles thus far known to be responsible for FHS are dominant [6].

Overall, SRCAP appears to be a multifaceted protein implicated in several cellular processes, including chromatin regulation, transcription, and DNA repair [10, 18–22, 24, 27]. Therefore, investigating the cellular functions of SRCAP may provide clues to the genetic and molecular basis of FHS onset.

Here, we combined cell biology, reverse genetics, and biochemical approaches to study the subcellular localization of the endogenous SRCAP protein and assessed its involvement in cell cycle progression. Surprisingly, we found that SRCAP associates with components of the mitotic apparatus, including centrosomes, the spindle and midbody and its RNAi-mediated depletion in HeLa cells perturbs mitosis and cytokinesis. Importantly, SRCAP interacts at telophase with a number of cytokinesis regulators and positively controls their midbody recruitment. Similarly, DOM-A localizes to centrosomes and the midbody in *Drosophila* S2 cells, and its depletion results in cell division defects.

Together, our results provide first evidence suggesting that SRCAP plays previously undetected and evolutionarily conserved roles in ensuring proper cell division, independent of its functions in chromatin regulation. Moreover, our results emphasize a surprising scenario whereby alterations in cell division produced by SRCAP mutations may contribute to the onset of FHS.

## Results

### Unconventional subcellular localization of SRCAP during cell division

**Fig. 1:**
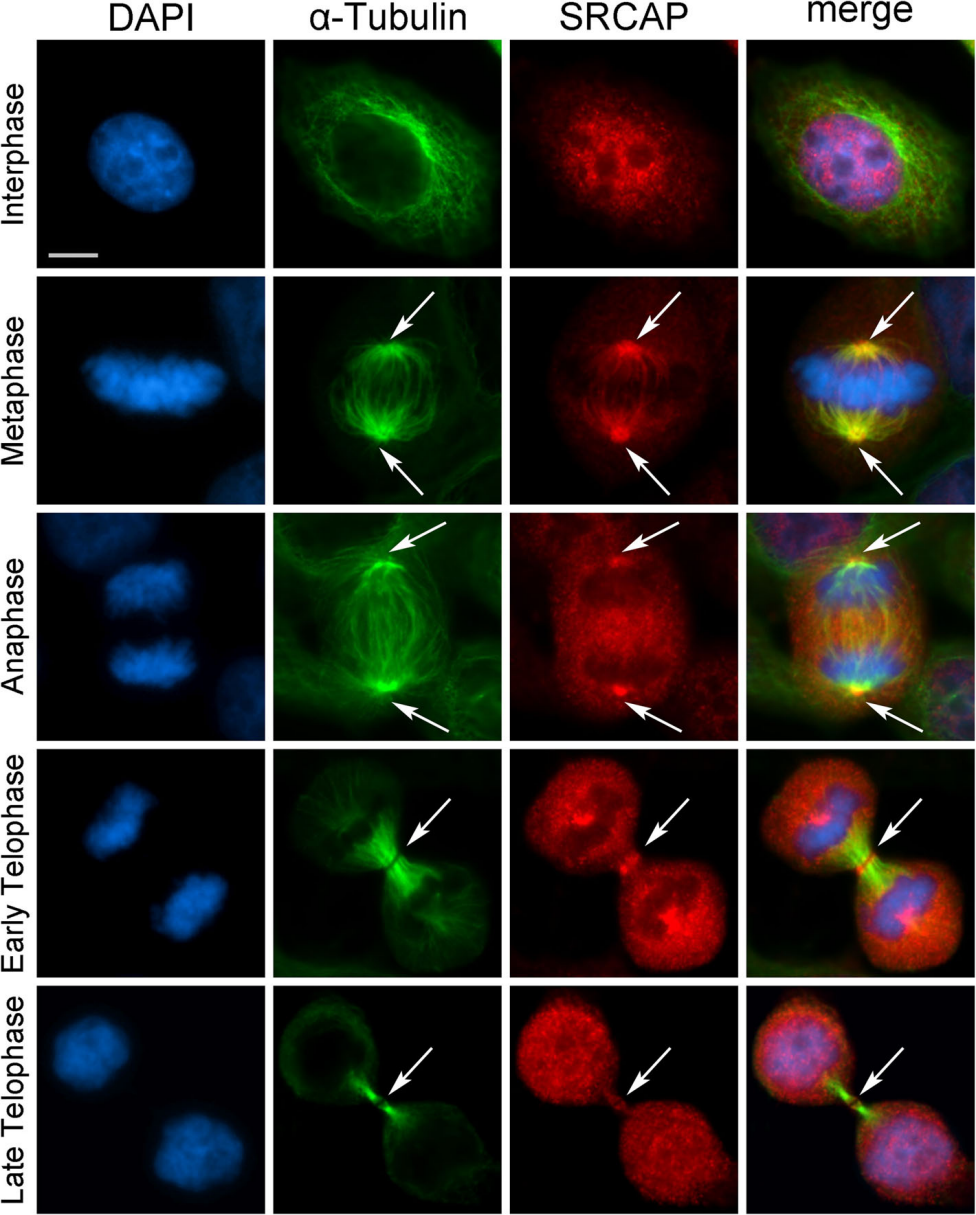
SRCAP localizes to the centrosomes, spindle, and midbody in HeLa cells. From left to the right: DAPI (blue), anti-α-Tubulin (green), anti-SRCAP (red) and merge. As expected, the SRCAP staining is present in the interphase nuclei. At metaphase, the SRCAP staining is found on spindle poles and spindle fibers, while in later stage decorates centrosomes and central spindle (anaphase) and midbody (telophase). Scale bar = 10 μm

First, we investigated the subcellular localization of the endogenous SRCAP protein during the cell cycle in HeLa cells using immunofluorescence microscopy (IFM). As shown in Fig. 1, a SRCAP polyclonal antibody (T15; Additional file 1: Table S1) decorated the interphase nuclei, as expected, but also revealed a specific pattern at the mitotic apparatus during mitotic progression. After nuclear envelope breakdown, SRCAP immunofluorescence redistributed at the mitotic spindle with enrichment at the poles and centrosomes and later at the central spindle and midbody.

The T15 antibody was validated by both IFM and Western blotting (WB) on HeLa cells transfected with a specific siRNA mix targeting SRCAP transcripts (see the “Methods” section). After SRCAP RNAi knockdown the antibody staining of nuclei, spindles and midbodies strongly decreased, as well as the amount of SRCAP protein present in the cells (Additional file 2: Fig. S1).

The subcellular localization of the endogenous SRCAP was confirmed in HuH7 hepatocyte carcinoma-derived cell line [28] and in human MRC5 fibroblast-derived cell line using the T15 antibody (Additional file 3: Fig. S2). In addition to the interphase nuclei, the antibody staining decorated centrosomes, spindle, and midbody, in line with the results in HeLa cells (Fig. 1). It then appears that the observed localizations of SRCAP reflect intrinsic properties of the protein, with no cell type specificity.

**Fig. 2:**
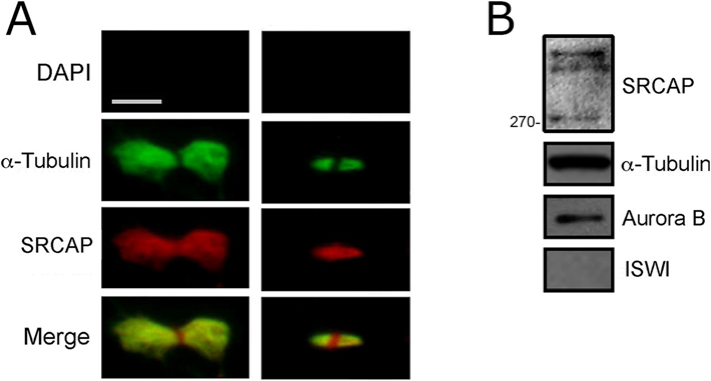
Localization of SRCAP on midbodies isolated from HeLa cells. Fixed preparations of midbodies were stained with DAPI (blue), anti-α-Tubulin (green), and anti-SRCAP antibody (red). **A** Immunolocalization of SRCAP protein to early (left) and late (right) midbodies. No DAPI staining was detected. SRCAP staining clearly decorated the isolated midbodies and overlapped with that of α-Tubulin. Scale bar = 5 μm. **B** Detection of SRCAP by Western blotting on protein extracts from isolated midbodies. Three high-molecular weight bands were detected (over 270 kD). These bands may be compatible with the three predicted SRCAP isoforms of 343, 337, and 327 kD. In fact, although proteins with minimal size differences should not be separated at high molecular weights, it is well known that the predicted molecular weight of a given protein not always corresponds to that found experimentally by SDS-PAGE. In the case of SRCAP isoforms, post-translational modifications may occur which could affect migration differences. Aurora B was used as a positive control. The ISWI remodeler (negative control) was not detected

The midbody is a tightly packed bridge that forms from the bipolar microtubule array derived from the anaphase central spindle. It serves as a platform for orchestrating cytokinesis by recruiting a large number of factors needed for abscission, the last stage of cell division [29]. Therefore, we wanted to evaluate the midbody association of SRCAP using both IFM and WB on isolated midbodies (see the “Methods” section). As shown in Fig. 2A, SRCAP immunofluorescence clearly decorated the isolated midbodies. WB analysis confirmed the presence of SRCAP in protein extracts from isolated midbodies (Fig. 2B). Taken together, these findings show that the subcellular localization of SRCAP is dynamic during cell division, in that it is recruited not only to interphase nuclei, but also to the centrosomes, spindle, and midbody. Remarkably, SRCAP is the core subunit of the homonymous complex governing H2A.Z deposition into chromatin [10, 18, 19], thus its association with the mitotic apparatus was not obvious.

### Depletion of SRCAP by siRNA-mediated knockdown perturbs mitosis and cytokinesis

**Fig. 3:**
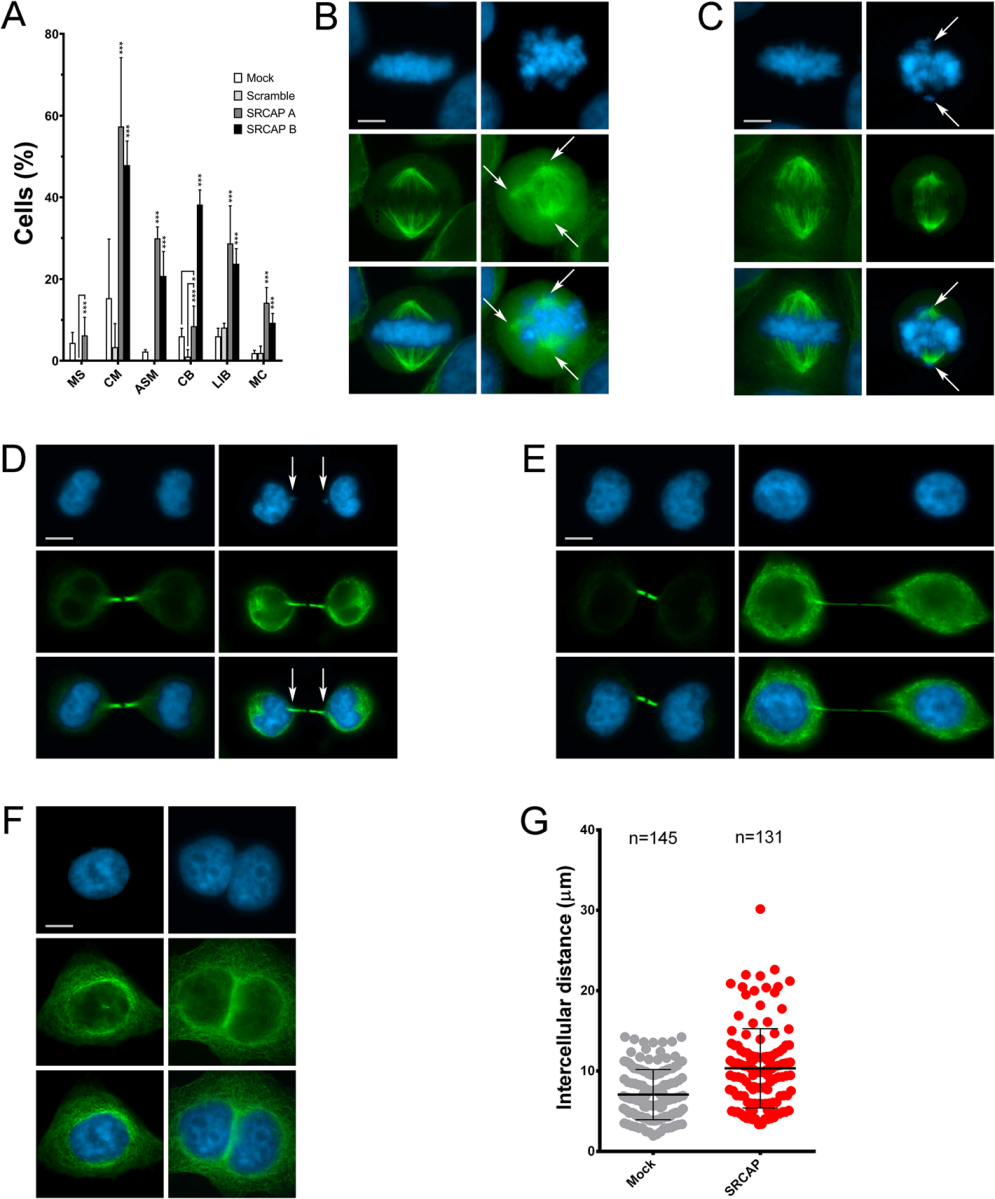
Depletion of SRCAP affects cell division in HeLa cells. RNAi knockdown was performed by transfecting HeLa cells with specific siRNAs (see the “Methods” section). Cells were stained with DAPI (blue) and anti-α-Tubulin (green). Left panels (mock), right panels (RNAi). Scale bar = 10 μm. Six classes of defects were categorized: **A** Histograms showing the quantitative analysis of cell division defects; mocks (white histograms), scramble (light gray histograms), SRCAP A (dark gray histograms), and SRCAP B (black histograms). **B** Multipolar spindles (MS). **C** Chromosome misalignments (CM) and abnormal spindle morphology (ASM). **D** Chromatin bridges (CB). **E** Long intercellular bridges (LIB); no DAPI-stained trapped chromatin was observed. **F** Multinucleated cells (MC). **G** Intercellular distance. The quantitative analysis of defects scored in RNAi-treated and control cells (Table 1) is based on the following numbers: at least 100 prometaphases and metaphases for MS, 70 metaphases for CM and ASM, 300 telophases for LIB and CB, and 5500 for MS. Three independent experiments were performed. *P < 0.05; **P < 0.005; and ***P < 0.0005 compared with the controls group (mock and scramble) by Fisher’s exact test

Next, we examined the functional significance of SRCAP recruitment to centrosomes, the spindle, and midbody by investigating the progression of cell division in SRCAP-depleted HeLa. Depletion of SRCAP was performed by transfecting Hela cells with specific siRNAs targeting SRCAP transcripts (see the “Methods” section): SRCAP A (mix of two oligos from Sant Cruz Biotechnology) and SRCAP B (a single oligo). As negative controls, samples were transfected with a scrambled oligo or processed excluding the addition of siRNAs (see the “Methods” section). In fixed HeLa cell preparations, we categorized and quantified six classes of cell division defects (Fig. 3A–G and Table 1): multipolar spindles (MS) at pro-metaphase and metaphase (Fig. 3B), chromosome misalignments (CM) and altered spindle morphology (ASM) at metaphase (Fig. 3C), chromatin bridges (CB) at anaphase and telophase (Fig. 3D), long thin intercellular bridges (LIB) at the last stage of telophase (Fig. 3E), and multinucleated cells (MC) (Fig. 3F). Compared to mock- and scramble-treated control cells, SRCAP RNAi-treated cells exhibited a significant increase in mitosis and cytokinesis defects. The increase of MS was only observed with SRCAP B compared to the scramble. The increase was particularly relevant for CM, where the misaligned chromosomes carry the centromere (Additional file 4: Fig. S3), strongly suggesting that they were not lost fragments resulting from chromosome breaks. SRCAP-depleted cells also exhibited a significant amount of abnormally shaped spindles (ASM), shorter and thinner than those of the control cells (Figs. 3A, C). A strong increase of CB was found only using the SRCAP B siRNA. Moreover, a relevant increase of LIB was observed. A LIB is defined as overextended, stretched, intercellular bridge that forms as a consequence of a failure of abscission, the final stage of cytokinesis. Consistently, the intercellular distance at the abscission stage in SRCAP-depleted cells was enhanced compared to control cells (Fig. 3G). Defective cytokinesis was also reflected in the appearance of MC. Thus, it appears that SRCAP depletion in HeLa cells disrupts both mitosis and cytokinesis, suggesting that the localizations observed at centrosomes, the spindle, and midbody reflect its functional roles in cell division.

**Table 1:** Cell division defects found in SRCAP depleted HeLa cells

		Control		RNAi	
		Mock (%)	Scramble (%)	SRCAP A (%)	SRCAP B (%)
Metaphase	MS	4.38 ± 2.58	0	6.21 ± 4.43 ***(scramble)	0
	CM	15.31 ± 14.45	3.30 ± 5.80	57.36 ± 16.78***	47.90 ± 5.90***
	ASM	2.25 ± 0.45	0	29.93 ± 2.80***	20.8 ± 5.90***
Telophase	CB	6.00 ± 1.97	1.00 ± 1.70	8.45 ± 4.96 *(mock), ***(scramble)	38.20 ± 3.60***
	LIB	1.87 ± 0.67	8.10 ± 1.10	28.69 ± 9.21**	23.80 ± 3.60***
	MC	2.16 ± 0.49	1.90 ± 1.70	14.21 ± 3.74***	9.30 ± 2.30***

CB, chromatin bridges; CM, chromosome misalignments; LIB, long intercellular bridges; MC, multinucleated cells; MS, multipolar Spindles

The results are expressed as mean ± SD values from three independent replicate experiments: *P < 0.05; **P < 0.005; and ***P < 0.0005 compared with the controls group by Fisher’s exact test

Notably, MS found in cells treated with SRCAP A are statistically significant (***) compared to scramble, but not to mock. CB show different statistically significant levels depending on comparison between SRCAP A and scramble (***) or mock (*)

### SRCAP depletion affects spindle microtubule repolymerization

**Fig. 4:**
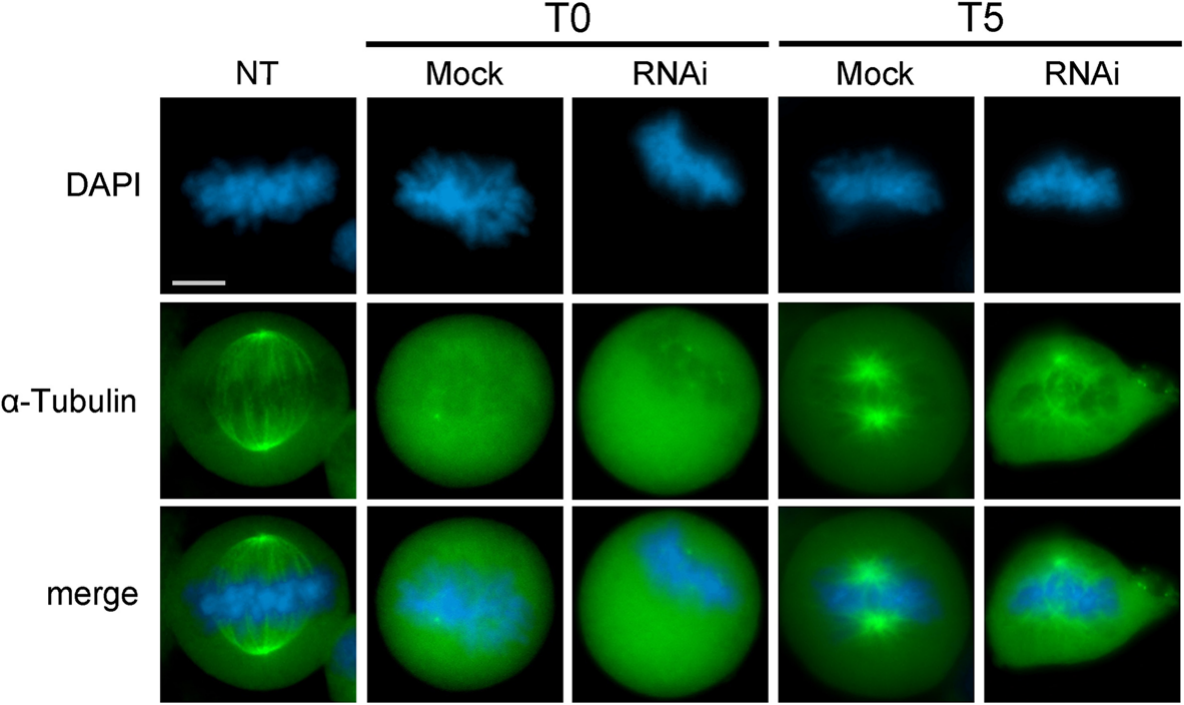
Abnormal microtubules re-polymerization in SRCAP depleted HeLa cells. From top to bottom: DAPI (blue), EGFP::α-Tubulin (green) and merge. Hela cells were incubated in ice (1 h) to stimulate microtubules depolymerization (T0). Compared to the non-treated samples (NT), microtubule re-polymerization after 5 min (T5) of rewarming give rise to properly shaped asters in control metaphases (mock), while in SRCAP depleted metaphases (RNAi), aster reformation is clearly aberrant with EGFP::α-Tubulin fluorescence marking only one pole spot, together with sparse and disorganized fibers. The results are based on a total of three experiments; at least 300 cells were scored from both RNAi-treated and control cells. Scale bar = 10 μm

The finding that SRCAP depletion affected spindle shape and chromosome alignment at metaphase (Fig. 3A, C) prompted us to investigate an involvement of this protein in the regulation of microtubule organization and mitotic spindle formation. Then, we used HeLa cells stably expressing EGFP::α-Tubulin and assessed whether SRCAP depletion influences microtubule regrowth after cold-induced disassembly. Control (mock) and SRCAP RNAi-depleted HeLa cells (RNAi) were incubated on ice for 1 h to induce extensive depolymerization (T0). The cells were then allowed to rewarm at 37 °C in complete medium for 5 min (T5) to resume microtubule regrowth. As shown in Fig. 4, microtubule re-polymerization after 5 min of rewarming resulted in clearly aberrant asters with rare, long, and thin MTs in RNAi-treated (mean ± SD, 36.84% ± 3.76) compared to the mock-treated cells (mean ± SD 5% ± 1.92). This result supports a role of SRCAP protein in microtubule organization and mitotic spindle assembly.

### SRCAP-dependent localization of cytokinesis regulators to the midbody

**Fig. 5:**
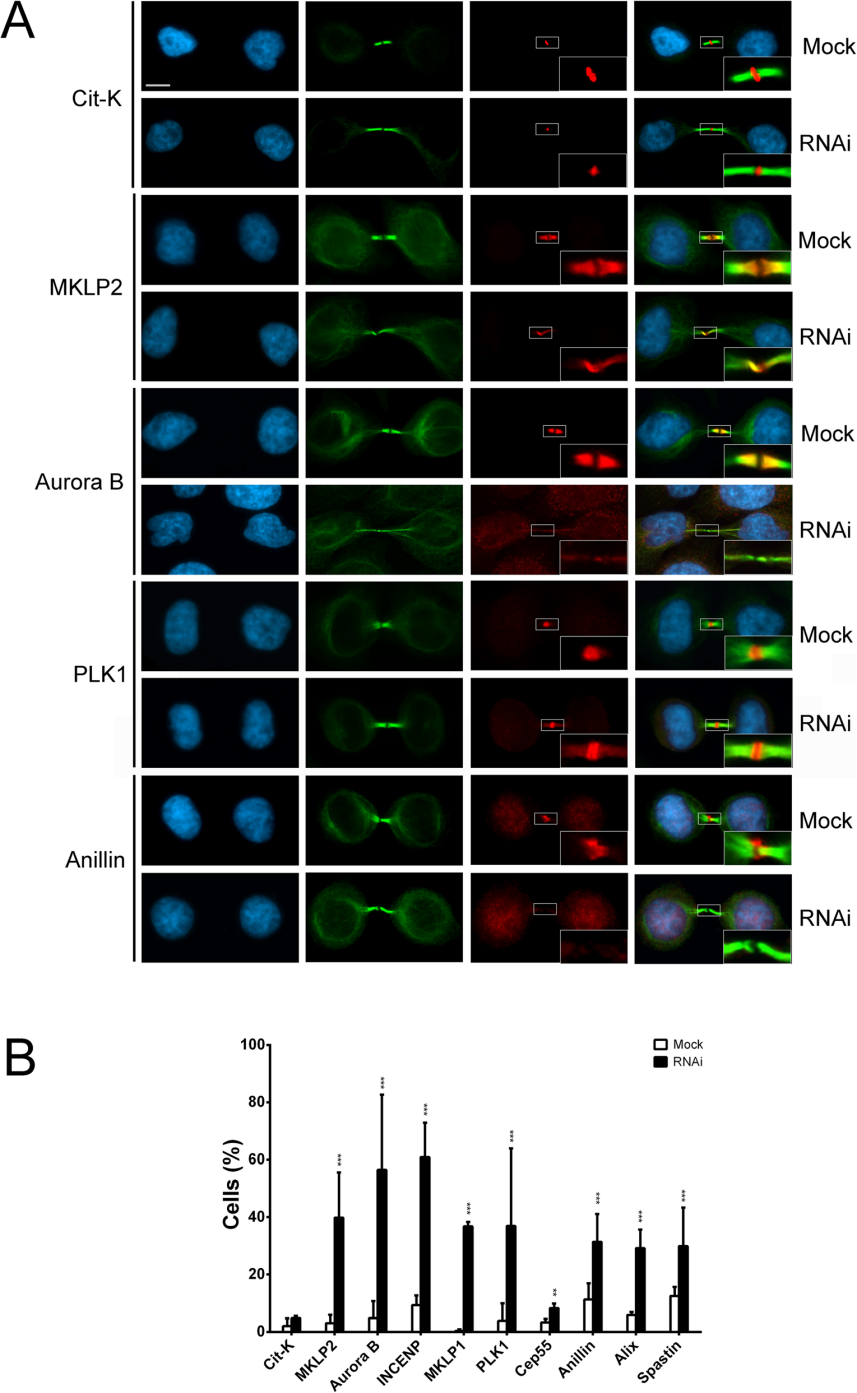
SRCAP depletion affects midbody localization of cytokinesis regulators. **A** Examples of cytokinesis regulators recruitment at midbody in mock and SRCAP depleted HeLa cells (RNAi). From left to the right: DAPI (blue), anti-α-Tubulin (green), cytokinesis regulators (red) and merge. **B** Histograms showing the quantitative analysis of mis-localizations (see also Table 2); mock (white histograms), SRCAP depleted cells (black histograms). Scale bar = 10 μm. Three independent experiments were performed and at least 300 telophases were scored in both RNAi-treated and control cells. *P < 0.05; **P < 0.005; and ***P < 0.0005 compared with the mock group by Fisher’s exact test

Cytokinesis is the last step of cell division and is controlled by a plethora of essential regulators recruited to the midbody during telophase [29–34]. The finding that SRCAP-depleted cultures are enriched in LIB and MC (Fig. 3) prompted us to investigate a possible role of SRCAP in cytokinesis. We used IFM to study the recruitment of crucial regulators of cytokinesis to the midbody in HeLa cells proficient or depleted in SRCAP. We focused on Cit-K, MKLP2, Aurora B, INCENP, MKLP1, PLK1, CEP55, Anillin, Alix, and Spastin, ten well-known proteins that localize to the midbody and are required for cytokinesis [29–34]. The results of three independent replicates shows that the midbody localization pattern of these factors was impaired in SRCAP depleted HeLa cells, with the exception of Cit-K (Fig. 5 and Table 2). For example, the midbody localization of Aurora B and Anillin was severely affected, while that of PLK1 became more widely distributed. These results suggested that SRCAP activity plays a role in the recruitment of a number of cytokinesis regulators to the midbody.

**Table 2:** Cytokinesis regulators mislocalization in SRCAP depleted HeLa cells

	Mock (%)	RNAi (%)
Cit-K	2.04 ± 2.65	4.80 ± 0.75
MKLP2	3.03 ± 2.90	39.84 ± 15.78***
Aurora B	4.81 ± 5.90	56.5 ± 26.16***
INCENP	9.28 ± 3.40	60.90 ± 11.93***
MKLP1	0.37 ± 0.57	36.78 ± 1.52***
PLK1	3.86 ± 6.06	36.91 ± 27***
Cep55	3.33 ± 1.15	8.27 ± 1.61**
Anillin	11.26 ± 5.66	31.38 ± .,67***
Alix	5.83 ± 1.06	29.15 ± 6.48***
Spastin	12.4 ± 3.18	29.90 ± 13.43***

The results are expressed as mean ± SD values from three independent replicate experiments: *P < 0.05; **P < 0.005; and ***P < 0.0005 compared with the mock group by Fisher’s exact test

### SRCAP interacts in telophase with cytokinesis regulators

**Fig. 6:**
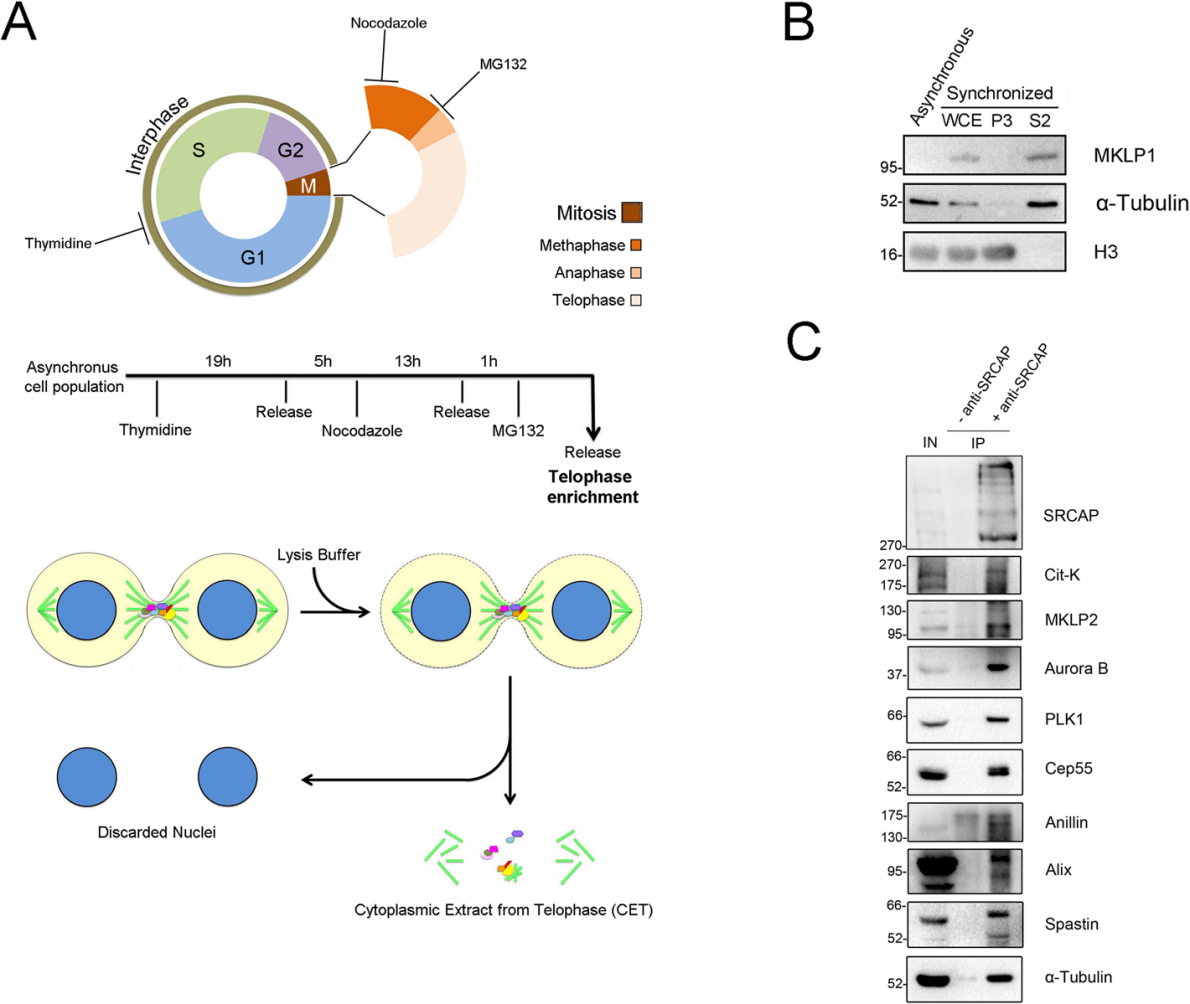
SRCAP interacts with cytokinesis regulators in co-IP assays. For immunoprecipitation assays, we used a SRCAP antibody previously validated by Ruhl et al. [9] (Additional file 5: Fig. S4 and Additional file 7: Table S1). **A** Telophase synchronization in HeLa cells. The scheme summarizes the protocol used for telophase synchronization and subcellular fractionation assay (see the “Methods” section). **B** Chromatin fractionation of HeLa cells synchronized in telophase. WCE, whole cell extract. P3, nuclear fraction. S2, cytoplasmatic fraction. H3 and α-Tubulin are markers of nuclear and cytoplasmic fraction, respectively. MKLP1 is expressed in late stages of mitosis (telophase synchronization control). **C** Immunoprecipitation of protein extracts from cytoplasmic fraction of telophase synchronized HeLa cells (S2 fraction). IP sample immunoprecipitated with SRCAP antibody (+ anti-SRCAP) were compared to negative control (- anti-SRCAP). Three independent IP experiments were performed. IN = input, IP = immunoprecipitation

The aforementioned results are also suggestive of possible interactions between SRCAP and cytokinesis regulators during telophase. To test this hypothesis, we carried out co-immunoprecipitation (co-IP) assays using an antibody previously validated by Ruhl et al. [9] (Additional file 5: Fig. S4, Additional file 8: Table S2) on protein extracts from the cytoplasmic fraction of telophase-synchronized HeLa cells (Fig. 6A, B; the “Methods” section). Synchronization was followed by subcellular fractionation assays to recover the cytoplasmic component (S2 fraction) and segregate away the chromatin-associated components. As shown in Fig. 6C, Cit-K, MKLP2, Aurora B, PLK1, CEP55, Anillin, Alix, and Spastin, together with α-Tubulin, were found in the IP sample immunoprecipitated with SRCAP antibody, but not in the negative control. Notably, the interaction between SRCAP and Anillin at midbody was highlighted by Capalbo et al. [35]. By contrast, MKLP1 and INCENP were not found in the IP (not shown). These results suggested that SRCAP interacts at midbody in telophase with essential cytokinesis regulators and with α-Tubulin, the main structural component of the midbody.

### Localization and RNAi-mediated depletion of DOM-A in Drosophila S2 cells

**Fig. 7:**
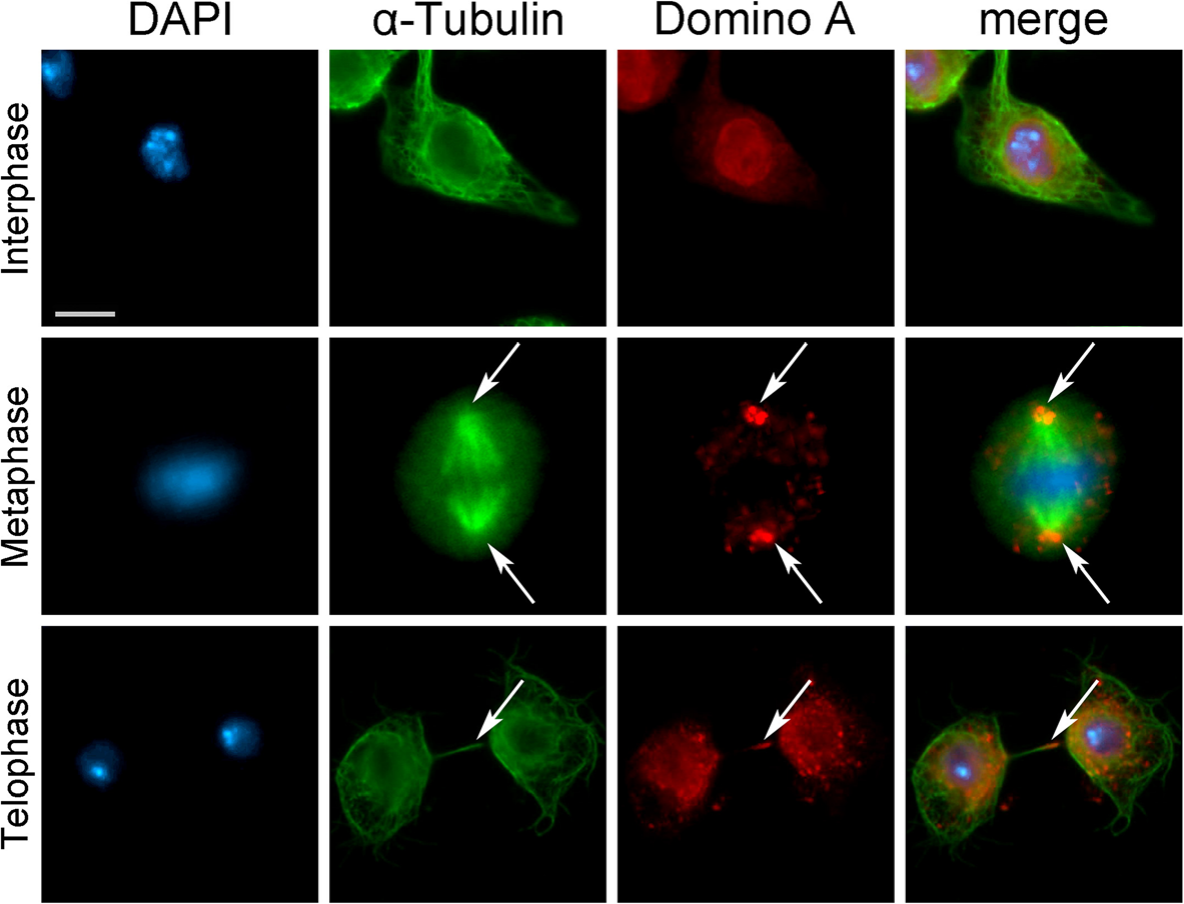
DOM-A localizes to interphase nuclei, centrosomes and midbody in *Drosophila* S2 cells. From left to the right: DAPI (blue), anti-α-Tubulin (green), anti-DOM-A (red) and merge. In addition to interphase nuclei, the anti-DOM-A staining was found on centrosomes (metaphase) and midbody (telophase) pointed by an arrow. Scale bar = 5 μm. *P < 0.05; **P < 0.005; and ***P < 0.0005 compared with the mock group by Fisher’s exact test

**Fig. 8:**
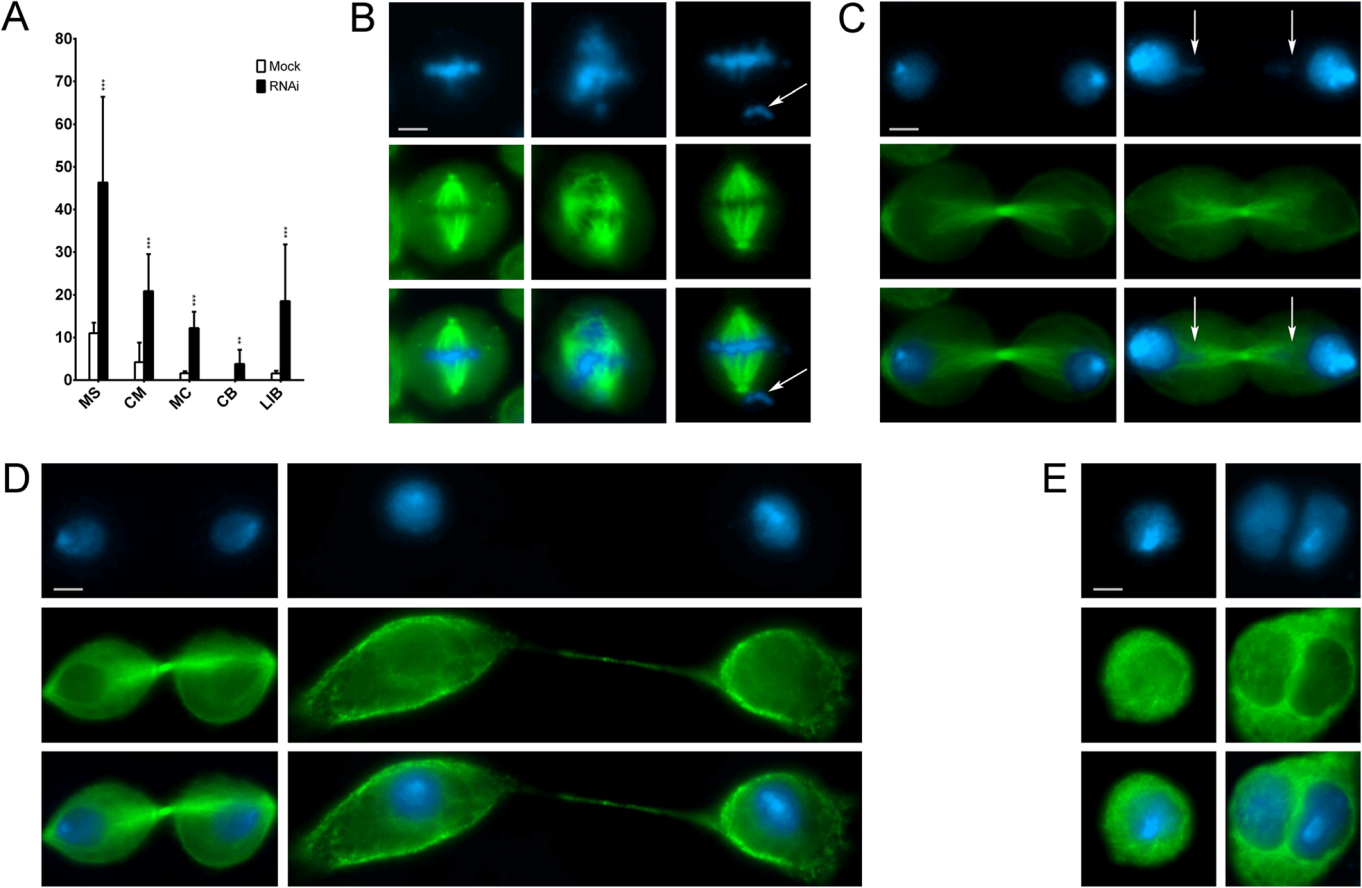
RNAi-mediated depletion of DOM-A affects mitosis and cytokinesis in *Drosophila* S2 cells. DAPI staining is shown in blue, α-Tubulin in green. **A** Quantitative analysis of defects; mock (white histograms), DOM-A depleted cells (black histograms). **B** From left to right: normal metaphase (mock), multipolar spindle (RNAi), and chromosome misalignments (RNAi). **C** Left panel: normal telophase (mock); right panel: chromatin bridge (RNAi). **D** left panel: normal telophase (mock); right panel: long intercellular bridge (RNAi). **E** Left panel: mononucleated cell (mock); right panel: binucleated cell (RNAi). Scale bar = 5 μm. The quantitative analysis of defects scored in RNAi-treated and mock treated cells is based on about 300 metaphases, telophases, or interphases, scored in at least three independent experiments (Table 3). *P < 0.05; **P < 0.005; and ***P < 0.0005 compared with the mock group by Fisher’s exact test

Lastly, we investigated whether the association of SRCAP with the mitotic apparatus and defects in cell division observed after its depletion are unique to human cells or are evolutionarily conserved. First, we used IFM to study the localization of DOM-A, the *Drosophila* ortholog of human SRCAP, in *Drosophila melanogaster* S2 cells. In addition to the interphase nuclei, a DOM-A antibody [23] decorated centrosomes and the midbody (Fig. 7). Next, we examined the phenotypes of S2 cells after RNAi against DOM-A. The RNAi efficiency was tested by sqRT-PCR and immunofluorescent assays (Additional file 6: Fig. S5), since the DOM-A antibody did not work properly for Western blotting under our conditions. Depletion of DOM-A resulted in mitotic phenotypes comparable to those observed in SRCAP-depleted HeLa cells (Fig. 8 and Table 3). Five categories of significant defects were observed: MS (46%), CM (21%), CB (4%), LIB (19%), and MC (12%). Importantly, these defects are consistent with the localization of DOM-A to centrosomes and the midbody in S2 cells.

**Table 3:** Cell division defects found in DOM-A depleted S2 cells

		DomA	
		Mock (%)	RNAi (%)
Metaphase	MS	11.01 ± 2.48	46.33 ± 20.14***
	CM	4.22 ± 4.58	20.9 ± 8.6***
Telophase	CB	0	3.81 ± 0.50**
	LIB	1.57 ± 0.6	18.54 ± 13***
	MC	1.16 ± 0.5	12.21 ± 3.84***

CB, chromatin bridges; CM, chromosome misalignments; LIB, long intercellular bridges; MC, multinucleated cells; MS, multipolar spindles

The results are expressed as mean ± SD values from three independent replicate experiments: *P < 0.05; **P < 0.005; and ***P < 0.0005 compared with the mock group by Fisher’s exact test

Next, we stained DOM-A-depleted S2 cells with an antibody against Spd2, a *Drosophila* centriole protein used as a centrosome marker [36]. We observed a high percentage of metaphase with multiple centrosomes exhibiting MT-nucleation ability, which gives rise to MS (Additional file 7: Fig. S6). Multiple centrosomes may arise from aberrant centriole disengagement/amplification, which in turn leads to the formation of MS and chromosome mis-segregation [37]. Alternatively, abnormal numbers of centrosomes can be a consequence of cytokinesis failure that results in the formation of MC.

## Discussion

Here, we showed that during cell cycle progression the ATPase SRCAP relocates to centrosomes and the spindle, and midbody, with its depletion yielding an array of aberrant outcomes of mitosis and cytokinesis (Figs. 1, 2, and 3). Similarly, DOM-A is found at centrosomes and the midbody in *Drosophila* S2 cells, and its depletion affects both mitosis and cytokinesis (Figs. 7 and 8). Moreover, SRCAP was found to interact at telophase with a number of cytokinesis regulators, positively controlling their recruitment to the midbody.

At least two alternative hypotheses can be considered to explain the defects found after SRCAP and DOM-A depletion: (1) the lack SRCAP or DOM-A may give rise to aberrant chromatin changes that alter the expression of genes involved in cell division and/or to perturbations in kinetochore and spindle organization and function. According to this hypothesis, the cell division defects caused by SRCAP and DOM-A depletion in HeLa and *Drosophila* S2 cells, respectively, are indirect and their recruitment to the mitotic apparatus only reflects a passive accumulation of disposable factors. (2) SRCAP and DOM-A proteins are essential components of the mitotic apparatus and participate in the control of cell division. Thus, their depletion is expected to directly affects mitosis and cytokinesis.

Several lines of evidence support the second hypothesis. Firstly, the recruitment of SRCAP and DOM-A to the mitotic apparatus, together with the disruption of specific steps of cell division caused by their depletion were observed in two distantly related species (common ancestor dates back to more than 700 million years ago). Such evolutionary conservation strongly suggests that SRCAP and DOM-A are essential components of mitotic apparatus.

Secondly, the observed defects do not appear to simply be a chaotic disruption of cell division, as one would expect by simultaneous upregulation or downregulation of genes encoding cell division regulators caused by chromatin perturbations. In contrast, SRCAP and DOM-A depletion leads to specific categories of mitosis and cytokinesis alterations. Notably, such alterations are consistent with the localization of SRCAP and DOM-A to the mitotic apparatus and also occur after the loss of crucial regulators of cell division [29–34].

Finally, and most importantly, a direct role of SRCAP in cytokinesis is supported by results indicating that it interacts with cytokinesis regulators in co-IP assays of chromatin-free protein extracts from telophase-synchronized HeLa cells (Fig. 6), with the midbody recruitment of the same regulators depending on SRCAP activity (Fig. 5 and Table 2). Notably, the interaction between SRCAP and Anillin has also been highlighted in a recent study on the midbody interactome [35]. Among the identified SRCAP interactors, Cit-K was not delocalized after SRCAP depletion (Fig. 5 and Table 2), suggesting that Cit-K may act in telophase upstream of SRCAP.

All of the proteins identified here as SRCAP interactors in telophase (Fig. 6) are midbody components essential for successful cell division in different organisms, as their depletion results in aberrant cytokinesis. Cit-K is the main abscission regulator capable of physically and functionally interacting with the actin-binding protein Anillin, a crucial component of the contractile ring and midbody [29, 38, 39]. MKLP2 is a motor kinesin that binds microtubules and is required for Aurora B recruitment to the central spindle [29, 40]. CEP55 recruits Alix at the midbody. Notably, in the absence of CEP55, a series of late-acting abscission factors fail to concentrate at the midbody, including Aurora B, MKLP2, PLK1, PRC1, and ECT2 [41], and the ESCRT machinery. Spastin is a key player in microtubule severing, ensuring the final cut at the midbody, whereas α-Tubulin is a major component of spindle and midbody microtubules.

### Unexpected roles of SRCAP in cell division

Our results suggest that SRCAP and DOM-A, similarly to other chromatin remodelers [42–50] are multifaceted proteins that, in addition to their canonical functions in interphase, play direct roles in mitosis and cytokinesis. In particular, cell division alterations (Fig. 3), spindle reformation defects (Fig. 4), and mis-localization of cytokinesis regulators at the midbody (Fig. 5) found in SRCAP-depleted cells, together with specific interactions detected in telophase-synchronized cells (Fig. 6) provides evidence that SRCAP participates in two different steps of cell division: (i) it may ensure proper chromosome segregation, regulating microtubule organization and mitotic spindle assembly, and (ii) it may be required for midbody function during abscission, acting as a platform for the recruitment of cytokinesis regulators and ensuring the final cut essential for proper abscission. In this context, we speculate that the ATPase activity of SRCAP could be required for its function in cell division. Indeed, several ATPases, such as Katanin, Cdc48/p97, ISWI, VPS4, and Spastin, interact with microtubules and play direct roles in mitosis and cytokinesis [51–53]. Intriguingly, depletion of Spastin at midbody results in cytokinesis failure phenotypes similar to those found in SRCAP-depleted cells [54].

## Conclusions

In conclusion, our results reveal the existence of a previously undetected and evolutionarily conserved phenomenon, whereby SRCAP is recruited to the mitotic apparatus during cell cycle progression in human cell lines (Fig. 1, Fig. 2, Additional file 3: Fig. S2) and has functional relevance in cell division preventing genetic instability. Therefore, we propose that mitosis and cytokinesis failure may contribute to the onset of developmental defects characteristic of FHS.

It is well known that defective mitosis or cytokinesis can cause chromosomal instability leading to genetically unstable states, hence activating tumorigenic transformation [55, 56]. A first case of a tumor associated with FHS has indeed been reported in 2009 [57]. Thus, it might be possible that FHS patients also exhibit some predisposition for tumor development. If this was true, then FHS patients should be subjected to clinical trials for cancer prevention.

HeLa cells are a system of election to study cell division due to a number of characteristics which have enabled very powerful, extensive genome-wide screening for mitotic genes [29, 55, 58]. However, it will be important to reassess in the future the findings herein obtained and validate the results switching to other cell types such as fibroblasts or lymphoblastoid cell lines from FHS patients.

## Methods

### Cytology and immunostaining

Cytology and immunostaining of human cell lines and *Drosophila melanogaster* S2 cells were performed according to Messina et al. [15] and Somma et al. [59], respectively.

### Cell cultures, transfections, and RNAi treatments

HeLa cells (ATTC company) were cultured in 6-well plates in Dulbecco’s modified Eagle’s medium (DMEM) supplemented with 10% FBS (Corning) and a penicillin/streptomycin solution (Gibco, 15140122). RNAi-mediated depletion of SRCAP was performed by double transfection (24 h + 48 h after seeding) with (i) a specific siRNA mix called SRCAP A (CCAGUUCCCUGACUUAAGATT + GGAUGGAUCUACUAGAGUUTT) targeting SRCAP transcripts at sequences CCAGTTCCCTGACTTAAGA and GGATGGATCTACTAGAGTT (sc-93293, Santa Cruz Biotechnology) and (ii) a single siRNA called SRCAP B (GCGUGAUGUUGAACUGGGAGAUGGA) targeting SRCAP transcript at sequence GCGTGATGTTGAACTGGGAGATGGA, already validated by Moreno-Andres et al. [60]. As negative control, samples were processed in the same way, excluding the addition of siRNA. As additional control, we used a scrambled siRNA (CAUCGAGACGCUAGCAGAUCCUGCG), already validated by Moreno-Andres et al. [60]. The Lipofectamine RNAi-MAX reagent (Thermo Scientific) was used for transfections, according to the manufacturer’s protocol; 24 h after the second transfection, cells were harvested for cytological and immunoblotting analysis.

*Drosophila melanogaster* S2 cells were cultured at 25 °C in Schneider’s *Drosophila* Medium (Biowest). RNAi treatments were carried out according to Somma et al. [59]. To perform DOM-A depletion, each culture was inoculated with 15 μg of specific siRNA targeting the *domino* gene. Control samples were treated in the same way without addition of dsRNA. Both dsRNA-treated and control cells were grown for 96 h at 25 °C and then processed for either immunofluorescence or blotting analysis. To prepare dsRNA, individual gene sequences were amplified by PCR from genomic DNA obtained from first-instar larvae of a wild type *D. melanogaster* strain. The primers used in the PCR reactions were 48 nt base long and all contained a 5′ T7 RNA polymerase binding site (5′-GAATTAATACGACTCACTATAGGGAGAC-3′) joined to a DOM-A specific sequence. The sense and antisense gene-specific DOM-A primers were as follows: for-TCTGGTGCTCAGATCGTGTC; rev-GTTGTCTGCAGCACCTTCAA.

### sq-RT PCR

Total RNA was extracted from DOM-A-depleted Drosophila S2 cells and control, using Trizol reagent and retro-transcribed with Sensi-FAST cDNA synthesis kit (BioLine), according to manufacturer instructions. DOM-A specific bands were amplified with Hi-Fi Taq polymerase, normalized by housekeeping RpL32 levels and compared to the control. Primers used were: DOM-A (for – TAAAGCCGTCAGACCACGTC; rev – ATCGCTCATGGCTGCAAAAC) and RpL32 (for – GCCCAAGGGTATCGACAACA; rev – CTTGCGCTTCTTGGAGGAGA).

### Western blotting and immunoprecipitation

Western blotting was performed according to Messina et al. [15]. SRCAP protein immunoprecipitation was performed according to Messina et al. [15], using a rabbit polyclonal antibody against SRCAP (Kerafast company) validated by Ruhl et al. [9]. Cytosolic fraction (2 mg/ml) from subcellular fractionation assay (see the next paragraph) was used as input (IN). As negative control, no antibody was added to a same amount of IN and beads (Santa Cruz Biotechnology).

### Cell cycle synchronization and subcellular fractionation assay

For immunoprecipitation experiments, HeLa cells were synchronized in telophase using thymidine/nocodazole blocks. Cells were treated with 2 mM thymidine (Sigma, T9250) for 19 h, released from G1/S block in fresh media for 5 h, incubated with 40 nM nocodazole (Sigma, M1403) for 13 h, and harvested by mitotic shake-off. Mitotic cells were washed three times with PBS and released in fresh medium for 70′ before harvesting and freezing in liquid nitrogen. Telophase cells (2 × 10^7^) were prepared by resuspending in 1 mL of Buffer A for subcellular fractionation according to Messina et al. [14].

### Midbody isolation

The midbody association of SRCAP was also evaluated on isolated midbodies. Midbody isolation was performed according to McKenzie et al. [61]. IFM and Western blotting were performed by using the SRCAP T15 antibodies (Additional file 1: Table S1, Additional file 8: Table S2) as described in the above paragraphs.

### Microtubules re-polymerization assays

HeLa Kyoto EGFP-α-Tubulin/H2B-mCherry cell line (EMBL, Germany) were cultured and transfected according to the above section; 24 h after last transfection, cells were assayed for microtubules re-polymerization. Control (mock) and SRCAP RNAi-depleted cells (RNAi) were incubated 1 h in ice (T0) and then supplemented with complete medium for 5′ (T5) to resume microtubules polymerization at 37 °C. Asters length was evaluated for analysis using the ImageJ software.

### Antibodies

Primary antibodies and HRP-conjugated secondary antibodies used for IFM, WB, and IP experiments were described in Additional file 1: Table S1 and Additional file 8: Table S2, respectively.

### Microscope image acquisition

Both human and *Drosophila melanogaster* slides were analyzed using a computer-controlled Nikon Eclipse 50i epifluorescence microscope equipped with UV-1A EX 365/10 DM 400 BA 400, FITC EX 465-495 DM 505 BA 515-555 and TRITC EX 540/25 DM 565 BA 605/55 filters using Plan Achromat Microscope Objective 100XA/1.25 Oil OFN22 WD 0.2 objective and QImaging QICAM Fast 1394 Digital Camera, 12-bit, Mono. Images were imported into ImageJ software (http://rsbweb.nih.gov/ij/) and adjusted for brightness and contrast uniformly across entire fields where appropriate. The figures were constructed in Adobe Photoshop. Fluorescence intensity of SRCAP was assessed using the ImageJ software.

### Statistical analysis

Data analyses were performed using the GraphPad Prism software (GraphPad Software, Inc., La Jolla, CA, USA). All results are expressed as mean **±** SD values from three independent replicate experiments. *P* value lower than 0.05 (*P < 0.05) was considered to be statistically significant, using two-tailed Fisher’s exact test.

## Supplementary Information

**Additional file 1: Table S1.** List of primary antibodies (30 kb .xls)

**Additional file 2: Fig. S1.** Validation of SRCAP antibody (T15) by IFM and WB in SRCAP depleted cells. A) and B) The anti-SRCAP staining of nuclei, spindles and midbodies was strongly decreased (from 75% up to 90%) in SRCAP depleted cells (black histograms) compared to the mock (white histograms). Fluorescence intensity was measured using the ImageJ software. Scale bar = 10 μm. ***P < 0.0005, compared with the mock group by Fisher’s exact test. C) By WB, the T15 antibody detected three high-MW bands (over 270kD), the intermediate one is faint and can be appreciated at higher expositions (middle panel). These bands are compatible with the predicted SRCAP isoforms of about 343, 337 and 327kD (see also legend to Figure 2). The intensity of the two higher MW bands was decreased in SRCAP depleted cells (SRCAP RNAi) compared to the mock. The apparent lack of effect on the lower band could be due to a secondary structure assumed by the shorter *SRCAP* gene transcript affecting the siRNA binding, as already shown for other gene transcripts (Bohula et al., 2003; Luo and Chang., 2004). The ISWI chromatin remodeler was used as negative control. (300 kb .jpg)

**Additional file 3: Fig. S2.** Subcellular localization of endogenous SRCAP in HuH7 and MRC5 cell lines. Fixed HuH7 (A) and MRC5 (B) cells were stained with DAPI (blue), anti-α-Tubulin (green) and anti-SRCAP (red). At interphase, the SRCAP staining decorates the nuclei, while during metaphase and telophase it is found at centrosomes/spindle and midbody, respectively. Scale bar = 10 μm. (1,00 Mb .jpg)

**Additional file 4: Fig. S3.** IFM assays with CREST antibodies (kinetochore marker) on mock and SRCAP depleted metaphases. From top to bottom, DAPI (blue), anti-α-Tubulin (green), CREST (red) and merge. The analysis of 300 metaphases scored in three independent experiments showed that all misaligned chromosomes detected carry the centromere. Scale bar = 10 μm. (228 kb .jpg)

**Additional file 5: Fig. S4.** Validation of SRCAP antibody used for IP assays. The SRCAP polyclonal antibody (from Kerafast company; Additional file 1: Table S1) was already validated in IP assays by Ruhl et al., 2006. The antibody was tested by Western blotting on whole protein extracts from HeLa cells transfected with SRCAP B siRNA (see the “Methods” section), compared to control samples (100%, 50% or 25%). High-molecular weight bands were detected over 270 kD, most of which are decreased in SRCAP depleted cells (SRCAP RNAi). The vinculin was used as negative control. (131 kb .jpg)

**Additional file 6: Fig. S5.** Efficiency of RNAi-mediated depletion of DOM-A protein in Drosophila S2 cells. After RNAi treatments with specific DOM-A siRNAs, the decrease of DOM-A transcripts (A) and DOM-A protein at centrosomes (CS) and midbody (B) was measured by RT-PCR and IFM, respectively, and compared to control samples. Mock (white histograms), SRCAP depleted cells (black histograms). Fluorescence intensity on centrosomes (CS) and midbodies (MB) was assessed using the ImageJ software. The anti-DOM-A signal intensity is clearly decreased after DOM-A depletion compared to the mock. The results are based on three experiments; the sqRT-PCR product band of RNAi treated-cells was 66,14 ± 19 SD. *P < 0.05; **P < 0.005 and ***P < 0.0005, compared with the mock group by Fisher’s exact test. (150 kb .jpg)

**Additional file 7: Fig. S6.** Anti-Spd2 staining of DOM-A depleted and control cells. S2 cells were stained with DAPI (blue), anti-α-Tubulin (green) and anti-Spd2 (red). In DOM-A proficient cells (Mock), the anti-Spd2 staining is found on the two centrosomes at metaphase. In DOM-A depleted cells (RNAi), multiple Spd2 signals were found at multiple centrosomes, which nucleate microtubules of multipolar spindles. Scale bar = 5 μm. (368 kb .jpg)

**Additional file 8: Table S2.** Secondary antibodies list (20 kb .xls)

**Additional file 9: Raw data for Table 1.**

**Additional file 10: Raw data for Table 2.**

**Additional file 11: Raw data for Table 3.**

## Data Availability

All data generated or analyzed during this study are included in this published article and its supplementary information files.

## References

[1] Bickmore WA, van der Maarel SM (2003). Perturbations of chromatin structure in human genetic disease: recent advances. Hum Mol Genet.

[2] Bouazoune K, Kingston RE (2012). Chromatin remodeling by the CHD7 protein is impaired by mutations that cause human developmental disorders. Proc Natl Acad Sci U S A.

[3] Masliah-Planchon J, Bieche I, Guinebretiere JM, Bourdeaut F, Delattre O (2015). SWI/SNF chromatin remodeling and human malignancies. Annu Rev Pathol.

[4] Kumar R, Li DQ, Muller S, Knapp S (2016). Epigenomic regulation of oncogenesis by chromatin remodeling. Oncogene.

[5] White SM, Morgan A, Da Costa A, Lacombe D, Knight SJ, Houlston R, Whiteford ML, Newbury-Ecob RA, Hurst JA (2010). The phenotype of Floating-Harbor syndrome in 10 patients. Am J Med Genet A.

[6] Hood RL, Lines MA, Nikkel SM, Schwartzentruber J, Beaulieu C, Nowaczyk MJ, Allanson J, Kim CA, Wieczorek D, Moilanen JS, Lacombe D, Gillessen-Kaesbach G, Whiteford ML, Quaio CR, Gomy I, Bertola DR, Albrecht B, Platzer K, McGillivray G, Zou R, McLeod D, Chudley AE, Chodirker BN, Marcadier J, Majewski J, Bulman DE, White SM, Boycott KM, FORGE Canada Consortium (2012). Mutations in SRCAP, encoding SNF2-related CREBBP activator protein, cause Floating-Harbor syndrome. Am J Hum Genet.

[7] Nikkel SM, Dauber A, de Munnik S, Connolly M, Hood RL, Caluseriu O, Hurst J, Kini U, Nowaczyk MJ, Afenjar A (2013). The phenotype of Floating-Harbor syndrome: clinical characterization of 52 individuals with mutations in exon 34 of SRCAP. Orphanet journal of rare diseases.

[8] Messina G, Atterrato MT, Dimitri P (2016). When chromatin organisation floats astray: the Srcap gene and Floating-Harbor syndrome. J Med Genet.

[9] Ruhl DD, Jin J, Cai Y, Swanson S, Florens L, Washburn MP, Conaway RC, Conaway JW, Chrivia JC (2006). Purification of a human SRCAP complex that remodels chromatin by incorporating the histone variant H2A.Z into nucleosomes. Biochemistry.

[10] Clapier CR, Cairns BR (2009). The biology of chromatin remodeling complexes. Annu Rev Biochem.

[11] Bao Y, Shen X (2011). SnapShot: chromatin remodeling: INO80 and SWR1. Cell.

[12] Havugimana PC, Hart GT, Nepusz T, Yang H, Turinsky AL, Li Z, Wang PI, Boutz DR, Fong V, Phanse S, Babu M, Craig SA, Hu P, Wan C, Vlasblom J, Dar VUN, Bezginov A, Clark GW, Wu GC, Wodak SJ, Tillier ERM, Paccanaro A, Marcotte EM, Emili A (2012). A census of human soluble protein complexes. Cell.

[13] Messina G, Celauro E, Atterrato MT, Giordano E, Iwashita S, Dimitri P (2015). The Bucentaur (BCNT) protein family: a long-neglected class of essential proteins required for chromatin/chromosome organization and function. Chromosoma.

[14] Messina G, Atterrato MT, Fanti L, Giordano E, Dimitri P (2016). Expression of human Cfdp1 gene in Drosophila reveals new insights into the function of the evolutionarily conserved BCNT protein family. Sci Rep.

[15] Messina G, Atterrato MT, Prozzillo Y, Piacentini L, Losada A, Dimitri P (2017). The human Cranio Facial Development Protein 1 (Cfdp1) gene encodes a protein required for the maintenance of higher-order chromatin organization. Sci Rep.

[16] Prozzillo Y, Delle Monache F, Ferreri D, Cuticone S, Dimitri P, Messina G (2019). The true story of Yeti, the “Abominable” heterochromatic gene of Drosophila melanogaster. Front Physiol.

[17] Prozzillo Y, Cuticone S, Ferreri D, Fattorini G, Messina G, Dimitri P. In vivo silencing of genes coding for dTip60 chromatin remodeling complex subunits affects polytene chromosome organization and proper development in Drosophila melanogaster. Int J Mol Sci. 2021;22(9):4525. 10.3390/jims22094525. 10.3390/ijms22094525 PMC8123692 33926075

[18] Wong MM, Cox LK, Chrivia JC (2007). The chromatin remodeling protein, SRCAP, is critical for deposition of the histone variant H2A.Z at promoters. J Biol Chem.

[19] Feng Y, Tian Y, Wu Z, Xu Y (2018). Cryo-EM structure of human SRCAP complex. Cell Res.

[20] Greenberg RS, Long HK, Swigut T, Wysocka J (2019). Single amino acid change underlies distinct roles of H2A.Z subtypes in human syndrome. Cell.

[21] Johnston H, Kneer J, Chackalaparampil I, Yaciuk P, Chrivia J (1999). Identification of a novel SNF2/SWI2 protein family member, SRCAP, which interacts with CREB-binding protein. J Biol Chem.

[22] Dong S, Han J, Chen H, Liu T, Huen MSY, Yang Y, Guo C, Huang J (2014). The human SRCAP chromatin remodeling complex promotes DNA-end resection. Current biology : CB.

[23] Ruhf ML, Braun A, Papoulas O, Tamkun JW, Randsholt N, Meister M (2001). The domino gene of Drosophila encodes novel members of the SWI2/SNF2 family of DNA-dependent ATPases, which contribute to the silencing of homeotic genes. Development.

[24] Eissenberg JC, Wong M, Chrivia JC (2005). Human SRCAP and Drosophila melanogaster DOM are homologs that function in the notch signaling pathway. Mol Cell Biol.

[25] Kusch T, Florens L, Macdonald WH, Swanson SK, Glaser RL, Yates JR, Abmayr SM, Washburn MP, Workman JL (2004). Acetylation by Tip60 is required for selective histone variant exchange at DNA lesions. Science.

[26] Scacchetti A, Schauer T, Reim A, Apostolou Z, Campos Sparr A, Krause S, et al. Drosophila SWR1 and NuA4 complexes are defined by DOMINO isoforms. Elife. 2020;9. 10.7554/eLife.56325. 10.7554/eLife.56325 PMC7239659 32432549

[27] Monroy MA, Ruhl DD, Xu X, Granner DK, Yaciuk P, Chrivia JC (2001). Regulation of cAMP-responsive element-binding protein-mediated transcription by the SNF2/SWI-related protein, SRCAP. J Biol Chem.

[28] Nakabayashi H, Taketa K, Miyano K, Yamane T, Sato J (1982). Growth of human hepatoma cells lines with differentiated functions in chemically defined medium. Cancer Res.

[29] Normand G, King RW (2010). Understanding cytokinesis failure. Adv Exp Med Biol.

[30] Glotzer M (2005). The molecular requirements for cytokinesis. Science.

[31] Barr FA, Gruneberg U (2007). Cytokinesis: placing and making the final cut. Cell.

[32] Carlton JG, Caballe A, Agromayor M, Kloc M, Martin-Serrano J (2012). ESCRT-III governs the Aurora B-mediated abscission checkpoint through CHMP4C. Science.

[33] Hu CK, Coughlin M, Mitchison TJ (2012). Midbody assembly and its regulation during cytokinesis. Mol Biol Cell.

[34] Bassi ZI, Audusseau M, Riparbelli MG, Callaini G, D'Avino PP (2013). Citron kinase controls a molecular network required for midbody formation in cytokinesis. Proc Natl Acad Sci U S A.

[35] Capalbo L, Bassi ZI, Geymonat M, Todesca S, Copoiu L, Enright AJ, Callaini G, Riparbelli MG, Yu L, Choudhary JS, Ferrero E, Wheatley S, Douglas ME, Mishima M, D’Avino PP (2019). The midbody interactome reveals unexpected roles for PP1 phosphatases in cytokinesis. Nat Commun.

[36] Giansanti MG, Bucciarelli E, Bonaccorsi S, Gatti M (2008). Drosophila SPD-2 is an essential centriole component required for PCM recruitment and astral-microtubule nucleation. Current biology : CB.

[37] Marteil G, Guerrero A, Vieira AF, de Almeida BP, Machado P, Mendonca S, Mesquita M, Villarreal B, Fonseca I, Francia ME (2018). Over-elongation of centrioles in cancer promotes centriole amplification and chromosome missegregation. Nat Commun.

[38] Giansanti MG, Bonaccorsi S, Gatti M (1999). The role of anillin in meiotic cytokinesis of Drosophila males. J Cell Sci.

[39] Piekny AJ, Glotzer M (2008). Anillin is a scaffold protein that links RhoA, actin, and myosin during cytokinesis. Current biology : CB.

[40] Gruneberg U, Neef R, Honda R, Nigg EA, Barr FA (2004). Relocation of Aurora B from centromeres to the central spindle at the metaphase to anaphase transition requires MKlp2. J Cell Biol.

[41] Zhao WM, Seki A, Fang G (2006). Cep55, a microtubule-bundling protein, associates with centralspindlin to control the midbody integrity and cell abscission during cytokinesis. Mol Biol Cell.

[42] Sillibourne JE, Delaval B, Redick S, Sinha M, Doxsey SJ (2007). Chromatin remodeling proteins interact with pericentrin to regulate centrosome integrity. Mol Biol Cell.

[43] Gartner W, Rossbacher J, Zierhut B, Daneva T, Base W, Weissel M, Waldhausl W, Pasternack MS, Wagner L (2003). The ATP-dependent helicase RUVBL1/TIP49a associates with tubulin during mitosis. Cell Motil Cytoskeleton.

[44] Sigala B, Edwards M, Puri T, Tsaneva IR (2005). Relocalization of human chromatin remodeling cofactor TIP48 in mitosis. Exp Cell Res.

[45] Ducat D, Kawaguchi S, Liu H, Yates JR, Zheng Y (2008). Regulation of microtubule assembly and organization in mitosis by the AAA+ ATPase Pontin. Mol Biol Cell.

[46] Gentili C, Castor D, Kaden S, Lauterbach D, Gysi M, Steigemann P, Gerlich DW, Jiricny J, Ferrari S (2015). Chromosome missegregation associated with RUVBL1 deficiency. PLoS One.

[47] Corona DF, Langst G, Clapier CR, Bonte EJ, Ferrari S, Tamkun JW, Becker PB (1999). ISWI is an ATP-dependent nucleosome remodeling factor. Mol Cell.

[48] Yokoyama H, Rybina S, Santarella-Mellwig R, Mattaj IW, Karsenti E (2009). ISWI is a RanGTP-dependent MAP required for chromosome segregation. J Cell Biol.

[49] Zhang SM, Song M, Yang TY, Fan R, Liu XD, Zhou PK (2012). HIV-1 Tat impairs cell cycle control by targeting the Tip60, Plk1 and cyclin B1 ternary complex. Cell Cycle.

[50] Mo F, Zhuang X, Liu X, Yao PY, Qin B, Su Z, Zang J, Wang Z, Zhang J, Dou Z, Tian C, Teng M, Niu L, Hill DL, Fang G, Ding X, Fu C, Yao X (2016). Acetylation of Aurora B by TIP60 ensures accurate chromosomal segregation. Nat Chem Biol.

[51] Cao K, Nakajima R, Meyer HH, Zheng Y (2003). The AAA-ATPase Cdc48/p97 regulates spindle disassembly at the end of mitosis. Cell.

[52] Yang D, Rismanchi N, Renvoise B, Lippincott-Schwartz J, Blackstone C, Hurley JH (2008). Structural basis for midbody targeting of spastin by the ESCRT-III protein CHMP1B. Nat Struct Mol Biol.

[53] Joly N, Martino L, Gigant E, Dumont J, Pintard L (2016). Microtubule-severing activity of the AAA+ ATPase Katanin is essential for female meiotic spindle assembly. Development.

[54] Connell JW, Lindon C, Luzio JP, Reid E (2009). Spastin couples microtubule severing to membrane traffic in completion of cytokinesis and secretion. Traffic.

[55] Lens SMA, Medema RH (2019). Cytokinesis defects and cancer. Nat Rev Cancer.

[56] Ben-David U, Amon A (2020). Context is everything: aneuploidy in cancer. Nat Rev Genet.

[57] Nelson RA, McNamara M, Ellis W, Stein-Wexler R, Moghaddam B, Zwerdling T (2009). Floating-Harbor syndrome and intramedullary spinal cord ganglioglioma: case report and observations from the literature. Am J Med Genet A.

[58] Skop AR, Liu H, Yates J, Meyer BJ, Heald R (2004). Dissection of the mammalian midbody proteome reveals conserved cytokinesis mechanisms. Science.

[59] Somma MP, Fasulo B, Cenci G, Cundari E, Gatti M (2002). Molecular dissection of cytokinesis by RNA interference in Drosophila cultured cells. Mol Biol Cell.

[60] Moreno-Andres D, Yokoyama H, Scheufen A, Holzer G, Lue H, Schellhaus AK, Weberruss M, Takagi M, Antonin W (2020). VPS72/YL1-mediated H2A.Z deposition is required for nuclear reassembly after mitosis. Cells.

[61] McKenzie C, Bassi ZI, Debski J, Gottardo M, Callaini G, Dadlez M, et al. Cross regulation between Aurora B and Citron kinase controls midbody architecture in cytokinesis. Open Biol. 2016;6(3). 10.1098/rsob.160019. 10.1098/rsob.160019 PMC4821246 27009191

