# Supplementary figures and images for: The ATPase SRCAP is associated with the mitotic apparatus, uncovering novel molecular aspects of Floating-Harbor syndrome
Source: BMC Biol. 2021 Sep 2;19:184. doi: 10.1186/s12915-021-01109-x (PMC8414691; doi:10.1186/s12915-021-01109-x)

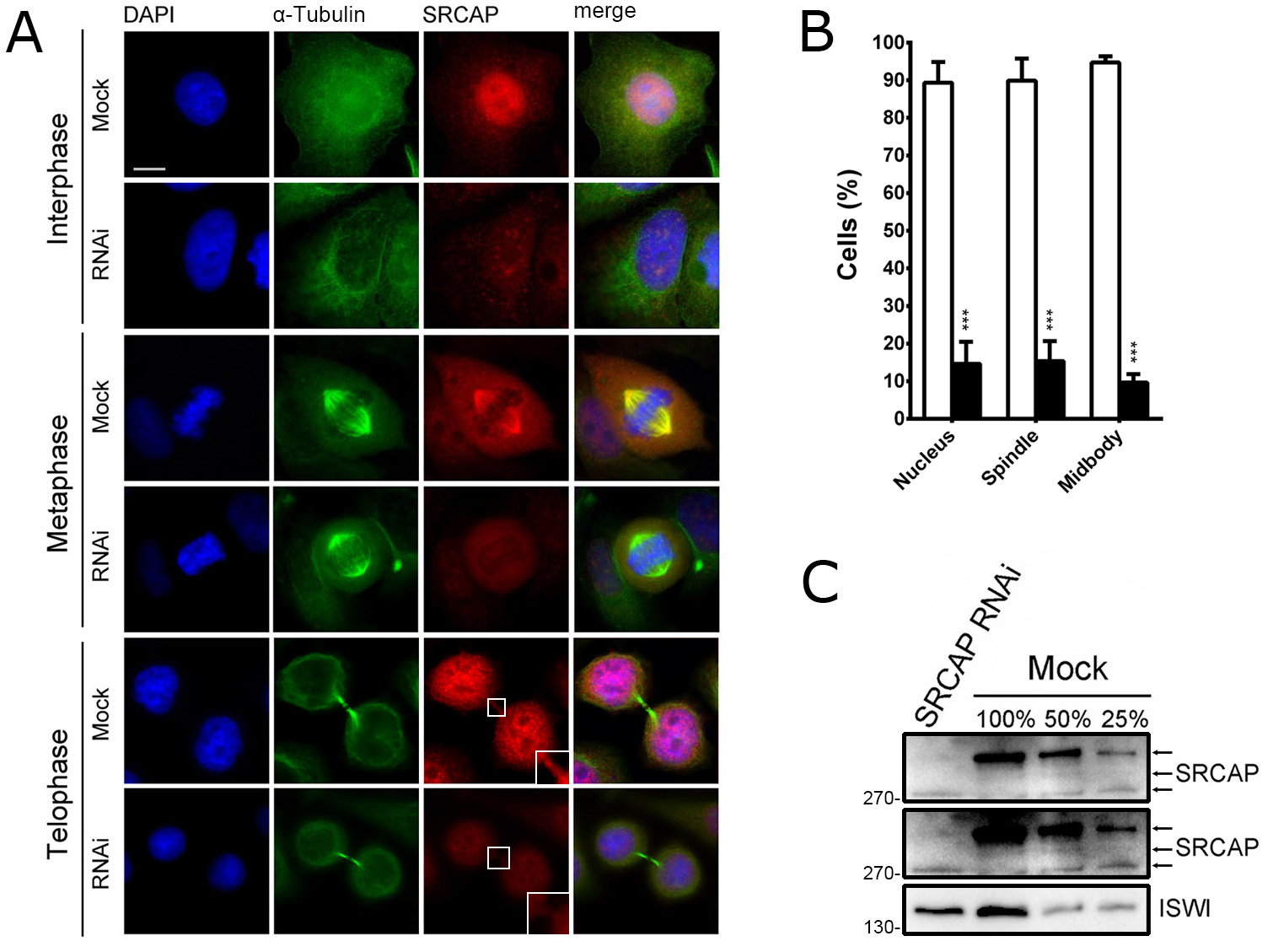

Supplement: Supplementary file 2 — Additional file 2: Fig. S1. Validation of SRCAP antibody (T15) by IFM and WB in SRCAP depleted cells. A) and B) The anti-SRCAP staining of nuclei, spindles and midbodies was strongly decreased (from 75% up to 90%) in SRCAP depleted cells (black histograms) compared to the mock (white histograms). Fluorescence intensity was measured using the ImageJ software. Scale bar = 10 μm. ***P < 0.0005, compared with the mock group by Fisher’s exact test. C) By WB, the T15 antibody detected three high-MW bands (over 270kD), the intermediate one is faint and can be appreciated at higher expositions (middle panel). These bands are compatible with the predicted SRCAP isoforms of about 343, 337 and 327kD (see also legend to Figure 2). The intensity of the two higher MW bands was decreased in SRCAP depleted cells (SRCAP RNAi) compared to the mock. The apparent lack of effect on the lower band could be due to a secondary structure assumed by the shorter SRCAP gene transcript affecting the siRNA binding, as already shown for other gene transcripts (Bohula et al., 2003; Luo and Chang., 2004). The ISWI chromatin remodeler was used as negative control. (300 kb .jpg) [file 12915_2021_1109_MOESM2_ESM.jpg]

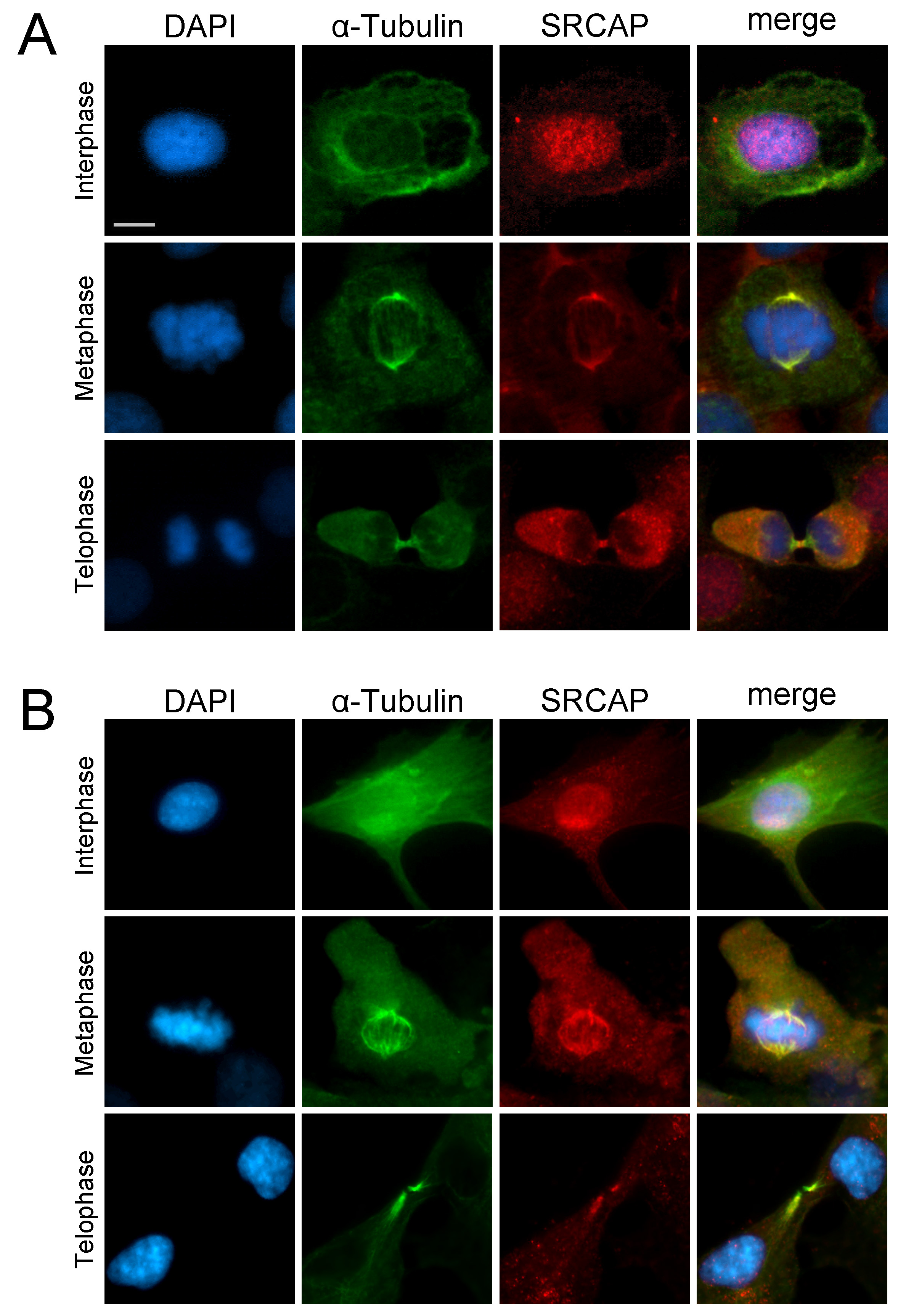

Supplement: Supplementary file 3 — Additional file 3: Fig. S2. Subcellular localization of endogenous SRCAP in HuH7 and MRC5 cell lines. Fixed HuH7 (A) and MRC5 (B) cells were stained with DAPI (blue), anti-α-Tubulin (green) and anti-SRCAP (red). At interphase, the SRCAP staining decorates the nuclei, while during metaphase and telophase it is found at centrosomes/spindle and midbody, respectively. Scale bar = 10 μm. (1,00 Mb .jpg) [file 12915_2021_1109_MOESM3_ESM.jpg]

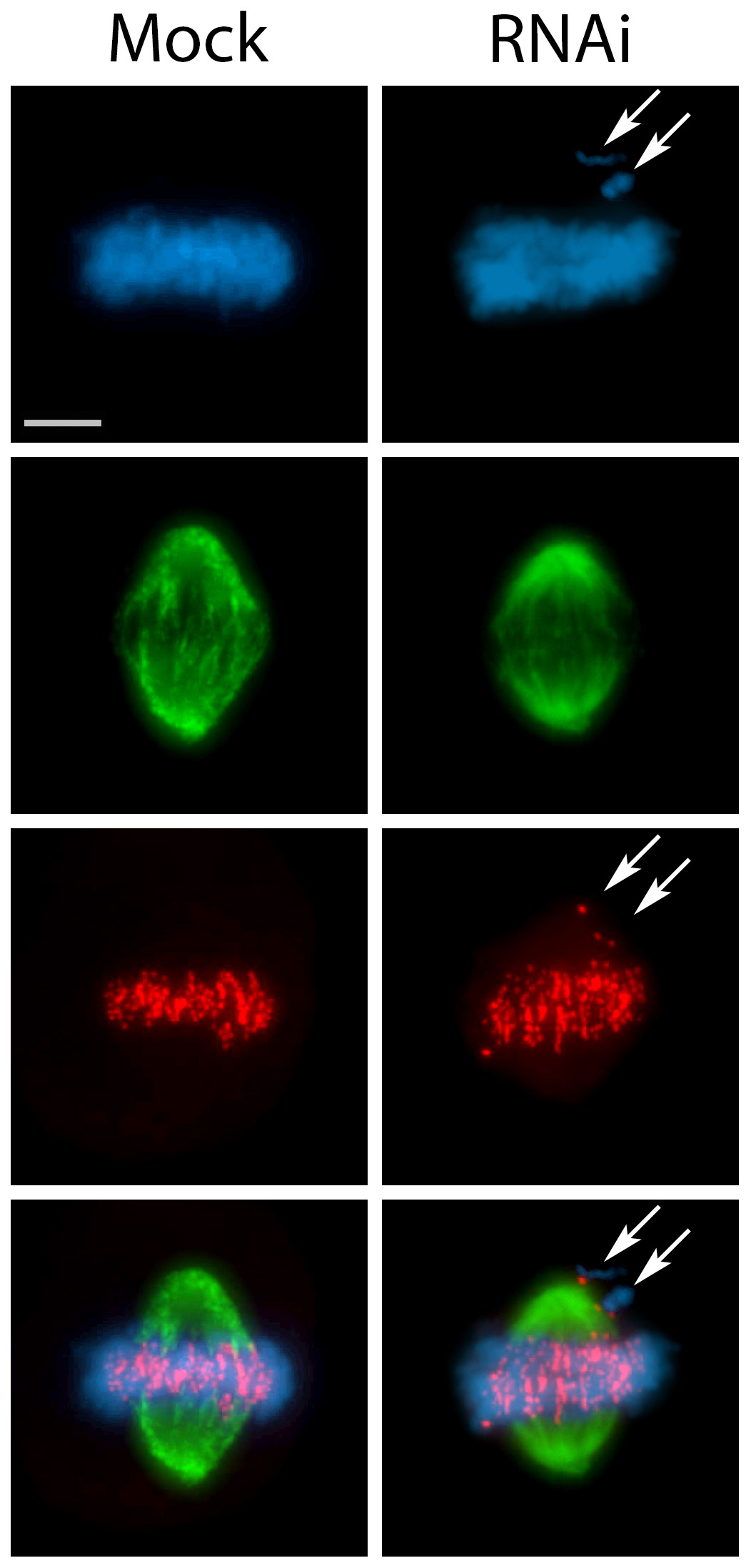

Supplement: Supplementary file 4 — Additional file 4: Fig. S3. IFM assays with CREST antibodies (kinetochore marker) on mock and SRCAP depleted metaphases. From top to bottom, DAPI (blue), anti-α-Tubulin (green), CREST (red) and merge. The analysis of 300 metaphases scored in three independent experiments showed that all misaligned chromosomes detected carry the centromere. Scale bar = 10 μm. (228 kb .jpg) [file 12915_2021_1109_MOESM4_ESM.jpg]

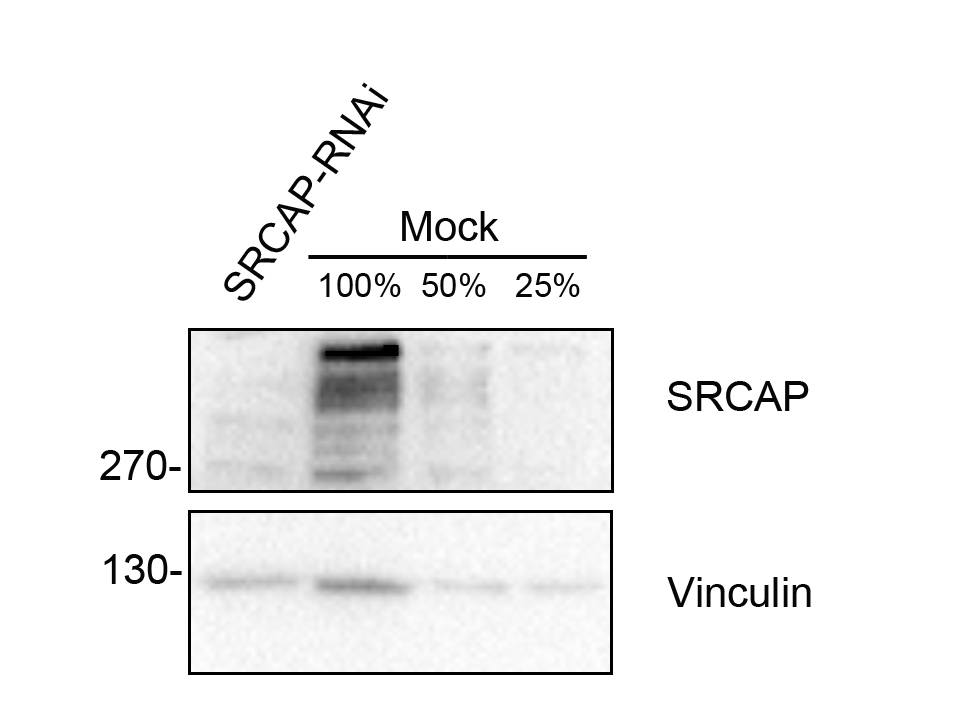

Supplement: Supplementary file 5 — Additional file 5: Fig. S4. Validation of SRCAP antibody used for IP assays. The SRCAP polyclonal antibody (from Kerafast company; Additional file 1: Table S1) was already validated in IP assays by Ruhl et al., 2006. The antibody was tested by Western blotting on whole protein extracts from HeLa cells transfected with SRCAP B siRNA (see the “Methods” section), compared to control samples (100%, 50% or 25%). High-molecular weight bands were detected over 270 kD, most of which are decreased in SRCAP depleted cells (SRCAP RNAi). The vinculin was used as negative control. (131 kb .jpg) [file 12915_2021_1109_MOESM5_ESM.jpg]

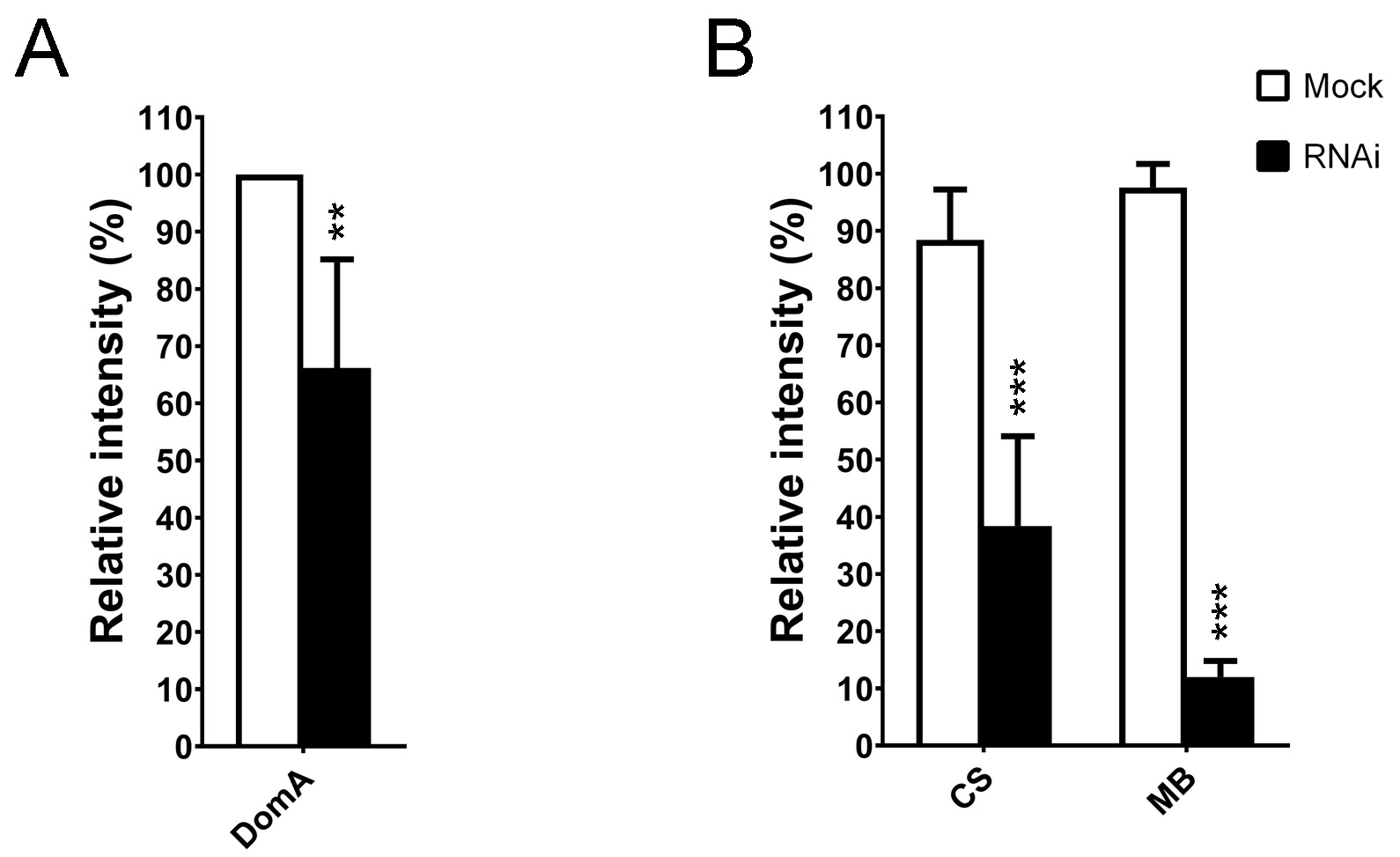

Supplement: Supplementary file 6 — Additional file 6: Fig. S5. Efficiency of RNAi-mediated depletion of DOM-A protein in Drosophila S2 cells. After RNAi treatments with specific DOM-A siRNAs, the decrease of DOM-A transcripts (A) and DOM-A protein at centrosomes (CS) and midbody (B) was measured by RT-PCR and IFM, respectively, and compared to control samples. Mock (white histograms), SRCAP depleted cells (black histograms). Fluorescence intensity on centrosomes (CS) and midbodies (MB) was assessed using the ImageJ software. The anti-DOM-A signal intensity is clearly decreased after DOM-A depletion compared to the mock. The results are based on three experiments; the sqRT-PCR product band of RNAi treated-cells was 66,14 ± 19 SD. *P < 0.05; **P < 0.005 and ***P < 0.0005, compared with the mock group by Fisher’s exact test. (150 kb .jpg) [file 12915_2021_1109_MOESM6_ESM.jpg]

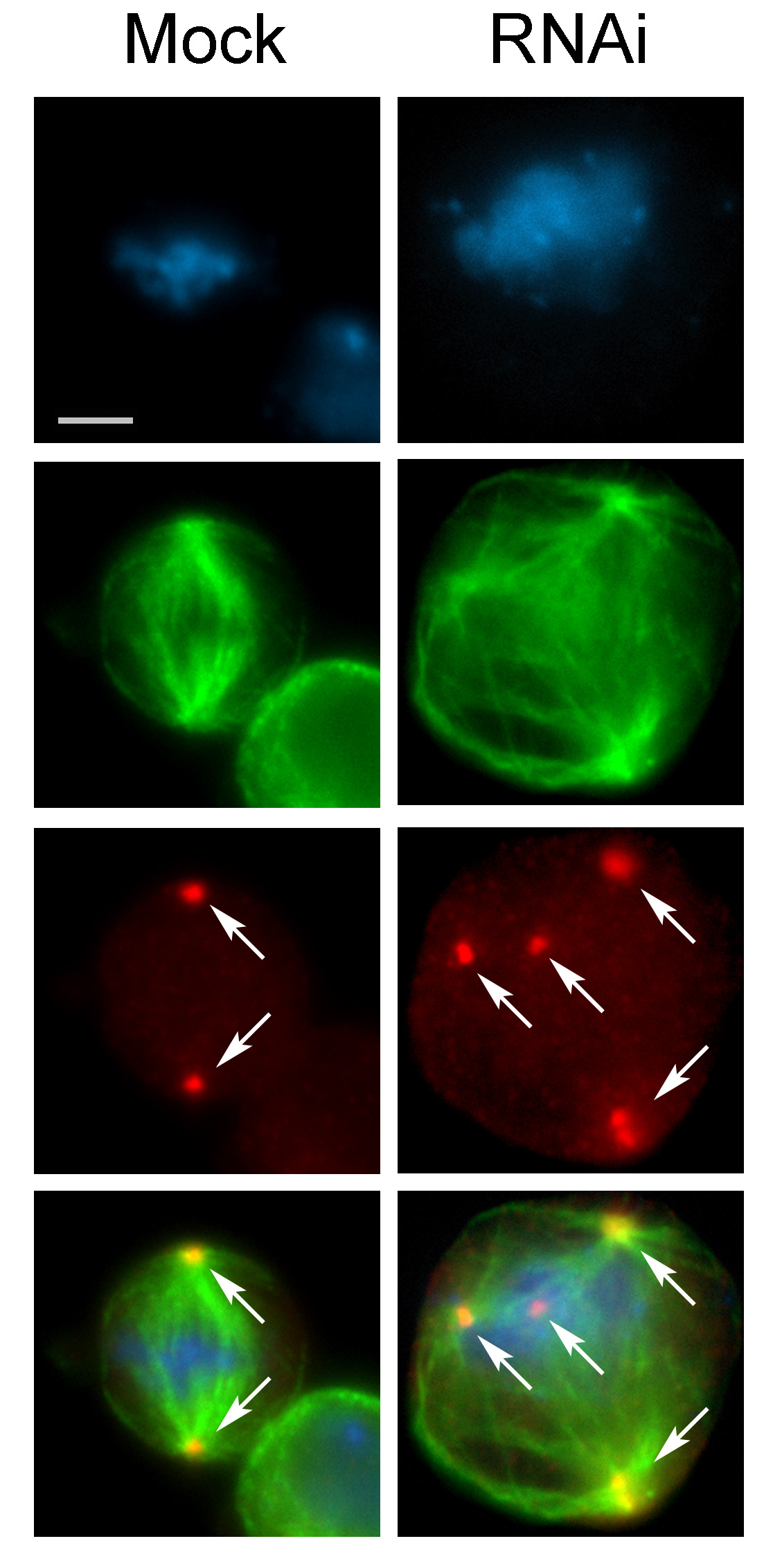

Supplement: Supplementary file 7 — Additional file 7: Fig. S6. Anti-Spd2 staining of DOM-A depleted and control cells. S2 cells were stained with DAPI (blue), anti-α-Tubulin (green) and anti-Spd2 (red). In DOM-A proficient cells (Mock), the anti-Spd2 staining is found on the two centrosomes at metaphase. In DOM-A depleted cells (RNAi), multiple Spd2 signals were found at multiple centrosomes, which nucleate microtubules of multipolar spindles. Scale bar = 5 μm. (368 kb .jpg) [file 12915_2021_1109_MOESM7_ESM.jpg]
